# Genome-wide identification and characterization of the *CKII* gene family in the cultivated banana cultivar (*Musa* spp. cv Tianbaojiao) and the wild banana (*Musa itinerans*)

**DOI:** 10.1371/journal.pone.0200149

**Published:** 2018-07-11

**Authors:** Weihua Liu, Zhengchun Lin, Yanying Liu, Yuling Lin, XuHan Xu, Zhongxiong Lai

**Affiliations:** Institute of Horticultural Biotechnology, Fujian Agriculture and Forestry University, Fuzhou, Fujian, China; Texas Tech University, UNITED STATES

## Abstract

Plant casein kinase II (CKII) plays an essential role in regulating plant growth and development, and responses to biotic and abiotic stresses. Here, we report the identification and characterization of the *CKII* family genes in *Musa* spp. cv. ‘Tianbaojiao’ (AAA group) and the wild banana (*Musa itinerans*). The 13 cDNA sequences of the *CKII* family members were identified both in ‘Tianbaojiao’ and wild banana, respectively. The differences between *CKII* α and *CKII* β members are corroborated through the subcellular localizations, phosphorylation sites and gene structures. The cloning of *CKII β-like-2* gDNA sequences in wild banana and ‘Tianbaojiao’ and the analysis of gene structures showed *MiCKIIβ-like-2b* and *MaCKIIβ-like-2* are likely alternatively spliced transcripts, which were derived from the alternative splicing events that involved exon deletion. The qPCR validation showed differential expression *CKII* family members in response to cold stress and also in all tested tissues (leaf, pseudostem and root) of wild banana. In particular, the normal transcript *MiCKIIβ-like-2a* was highly expressed in response to cold stress in wild banana; oppositely, the alternatively spliced transcript *MiCKIIβ-like-2b* was quite lowly expressed. The complex origin and long-term evolution of *Musa* lineage might explain the alternative splicing events of *CKII β-like-2*.

## Introduction

Casein kinase II (CKII or CK2) is a Ser/Thr kinase involved in the regulation of protein functions in eukaryotes. Plant CKII is a tetrameric protein composed of two catalytic (α) and two regulatory (β) subunits, and it is also a pleiotropic enzyme. It plays an essential role in regulating various cellular processes such as growth, development, circadian rhythms, light responses, hormone responses, transcription, translation, cell-cycle regulation, nuclear transport, Ca^2+^ storage, seed storage, salicylic acid-mediated defenses, flowering time, DNA repair and responses to biotic and abiotic stresses in plants, such as maize, tobacco, wheat, mustard and *Arabidopsis thaliana* [1–13]. Salinas et al (2006) [14] presented a complete survey of the *CKII* gene family and found four α subunits and four β subunit genes, which were all expressed in the inflorescences, stems, leaves and roots in *Arabidopsis*. Mulekar et al (2012) [4] further reported that *CKII* α subunits affect multiple developmental and stress-responsive pathways in *Arabidopsis*. Portoles and Mas (2010) [15] found that the functional interplay between *CKII* and *CCA1* (*circadian clock associated 1*) transcriptional activities is essential for clock temperature compensation in *Arabidopsis*. In plant cells, CKII is localized in the cytosol and the nucleus [16], and α subunits of the CKII family members are localized in the chloroplasts in mustard and *Arabidopsis* [8].

CKII is an extremely conserved pleiotropic protein kinase with more than 300 substrates [6,17]. The CKII phosphor acceptor sites are specified by multiple acidic residues, with the one at position +3 relative to the target residue being crucial. The CKII holoenzyme is composed of two catalytic subunits (αα, α’α’ or αα’), which act mainly as catalysts of phosphorylation, and a dimer of two non-catalytic β subunits, which act mainly as regulators of enzymatic activities [2,4,17]. Dennis and Browning (2009) reported the differential phosphorylation of plant translation initiation factors by *Arabidopsis thaliana* CKII holoenzymes [18]. Recent plant whole-genome sequencing projects will allow the precise structure and function of *CKII* to be full characterized.

Banana belongs to the genus *Musa*, a member of the family Musaceae, and is the most popular fruit in the worldwide. It is thermophilic crop, and distribute in the warm tropical or subtropical regions. Fujian province, in the northern margin of China is one such region prominent for banana cultivation. ‘Tianbaojiao’, which is the famous traditional cultivar in Fujian, often suffered low temperature stress in winter and early spring (S1 Fig). The critical temperature of growth is thought to be around 13°C for most banana cultivars in China [19]. The morphological changes of ‘Tianbaojiao’ leaves were quite different at low temperature stress (4°C) and when the treatment time was increased. The changes such as slight water logging (3 h), wilting (5–7 h) or death (at 28°C to recover) were observed (S2 Fig). The wild banana genetic resources are abundant in China, particularly in Fujian province. A novel wild banana line, which was found at Sanming city, Fujian province, is thought to be extremely cold resistant based on screening the wild banana genetic resources collected by our team for over 10 years [20]. It can grow well around 0°C [21], and its semilethal temperature was lower than other nine *Musa* genus plants, reached as low as -1.776°C [22]. So the wild banana (cold-resistant) and ‘Tianbaojiao’ (cold-sensitive) were used as materials to study the existence and expression of *CKII* family genes.

The banana genome has been published [23–24], allowing for the identification of *CKII* family genes in banana. In this study, the members of *CKII* family genes in banana genome A from *Musa acuminata* ‘DH-Pahang’ and in banana genome B from *Musa balbisiana* Pisang Klutuk Wulang (PKW) were analyzed using the banana genome’s data, and then the *CKII* gene family members in *Musa* spp. cv. ‘Tianbaojiao’ and the wild banana (*Musa itinerans*) were cloned and characterized, which is beneficial to understanding the structure and functions of the *CKII* family genes in *Musa* plant.

## Materials and methods

### 2.1 Analysis of the *CKII* family genes in banana genomes A and B

Using banana genome A (*Musa acuminata* var. DH-Pahang, AA group, the wild banana from Malaysia) [23] and banana genome B (*Musa balbisiana* var. Pisang Klutuk Wulang, BB group) [24] databases, the *CKII* genes were obtained by searching for the term of ‘casein kinase II’, and then the known *CKII* sequences in NCBI were used as the probes to searcher for the *CKII* genes in banana genomes. They were further analyzed as the candidate *CKII* genes of banana genomes.

### 2.2 Isolation of the *CKII* family genes cDNA sequences and *CKIIβ-like-2* gDNA sequences from wild banana and ‘Tianbaojiao’

The leaves of the wild banana (*Musa itinerans*) from Sanming City, China and the cultivated banana ‘Tianbaojiao’ (*Musa* spp., Cavendish, AAA group, the famous tranditional cultivar in China, which originated from the wild banana *Musa acuminate*, AA group) collected from the Banana Germplasm Nursery of Institute of Horticultural Biotechnology of Fujian Agriculture and Forestry University were used as the materials for RNA and DNA extraction, according to the method of Feng et al. (2015) [25]. Total RNA was reverse transcribed using a Thermo Scientific RevertAid First Strand cDNA Synthesis Kit (Fermentas, EU) for cDNA sequences cloning. Using candidate *CKII* genes in banana genomes combined with published *CKII* sequences in NCBI, all of the *CKII* family members cDNA sequences of wild banana and ‘Tianbaojiao’ were cloned by RT-PCR (reverse transcription PCR) technique. The *CKIIβ-like-2* gDNA sequences of wild banana and ‘Tianbaojiao’ were obtained from DNA templates using PCR. Primer sequences were designed from known *CKII* sequences in NCBI and the banana genome databases, and are listed in S1 Table.

### 2.3 Bioinformatic analysis of CKII family genes in the A genome, the wild banana and ‘Tianbaojiao’

On the NCBI website, the nucleotide and protein sequences of *CKII* family members were identified by BLASTn and BLASTp, respectively. DNAMEN 6.0 was used to analyze CDSs (coding sequences) and protein sequences [23–24]. MEGA 6.0 was used to construct the phylogenetic trees of the CKII proteins, and the NJ (neighbor-joining) method was then applied to this analysis with 1,000 bootstrap replications. GSDS was used to analyze the gene structures. The conserved domains were analyzed on the NCBI website. NetPhos 3.1 was used to analyze the CKII phosphorylation sites. The conserved motifs of CKII protein sequences were analyzed on MEME server [26]. The protein subcellular localization prediction tool of ‘PSORT’ was used to predict the subcellular location of the CKII protein sequences.

### 2.4 Plant materials and treatments for qPCR

The wild banana (*Musa itinerans*) from Sanming City and the cultivated banana ‘Tianbaojiao’ (*Musa* spp., AAA group) were used in this study. The *in-vitro* plantlets were regenerated by tissue culture from the explants of suckers. After transplanting them to the pots and cultivating for 1 month at 28°C under 2000 lx throughout lighting in a 12 h/12 h light-dark cycle, seedlings at the uniform growth stage were selected for treatments. After sufficiently watering for 2 d, the seedlings were put in the growth chambers set to 28°C (the control), and at 13°C, 4°C and 0°C, under 2000 lx fluorescent lighting in a 12 h/12 h light-dark cycle (synchronized with the natural light cycle) at a relative humidity of 70%-80% for 24 h. After 24 h treatments, the first young leaf was detached from 10 seedlings at each temperature point (28°C, 13°C, 4°C and 0°C) for each biological replicate. The leaf samples of each of the 10 seedlings were harvested and pooled for each temperature point. All of the treatments were performed with 3 biological replicates. Finally, they were frozen in liquid N_2_ and stored at -80°C for total RNA extraction and used in the qPCR (real-time quantitative PCR) assay. The leaves, roots, and pseudo-stems were also to taken from the potted plants grown at 28°C.

### 2.5 Real-time quantitative PCR and data analysis

The total RNA extracted from the leaves after cold treatments (including the control) using Column Plant RNAOUT 2.0 Kit (TIANDZ, China), and 0.5 ug total RNA was used for reverse transcription of qPCR analysis with PrimeScript™ RT Master Mix (Perfect Real Time) kit (Takara, Japan) according to the method of Feng et al. (2015) [25]. The expression detection of the *CKII* family genes was performed on a LightCycler 480 (Roche). The reaction system and procedures were those of Feng et al. (2015) [25]. The qPCR analyses were performed as described by Lin and Lai (2013) [27] and the *CAC* gene was used as the internal control [28]. The primer sequences were designed using Primer 3 input software and are listed in the S2 Table. The amplification efficiency for each primer pairs of the *CKII* family genes was determined in a qPCR assay using a five-fold dilution series from a pooled cDNA template (S3 Fig). The PCR efficiency values of all *CKII* family genes ranged from 1.853 to 2.040, and as listed in S2 Table. SPSS was used to assess the statistically significant differences of data, and all data are expressed as the means ± SDs of three independent replicates. Duncan’s multiple range test was used for the significant differences. *: significant difference (at p-value <0.05) identified by comparing with 28°C, **: very significant difference (at p-value <0.01) identified by comparing with 28°C.

## Results

### 3.1 Analysis of the *CKII* family genes in banana genomes A and B

In total, there are 13 *CKII* family genes in banana genome A and 11 *CKII* family genes in banana genome B. The functional domains analyses indicated that all 13 members of the *CKII* family in banana genome A contained STKc_CK2_alpha or CK_II_beta functional domains, while the 11 *CKII* family genes of banana genome B had only three members (ITC1587_Bchr2_P04995, ITC1587_Bchr6_P16283 and ITC1587_Bchr6_P18330) that contained complete functional domains (STKc_CK2_alpha or CK_II_beta). The phylogenetic tree of the 13 CKII members from genome A and the 11 CKII members from genome B had two branches, one containing CKII α members and the other containing CKII β members and unclassified CKII subunits members. Additionally, ITC1587_Bchr9_P25856 and ITC1587_Bchr5_P13181, with PKc-like superfamily domains, were clustered to a clade (S4 Fig). Thus, the integrity and accuracy of *CKII* from genome A was greater than that from genome B, which was suggested to function as the reference genome for identification of the *CKII* gene family members in the genus *Musa*.

### 3.2 Cloning of the *CKII* gene family members in ‘Tianbaojiao’ and the wild banana

**Fig 1:**
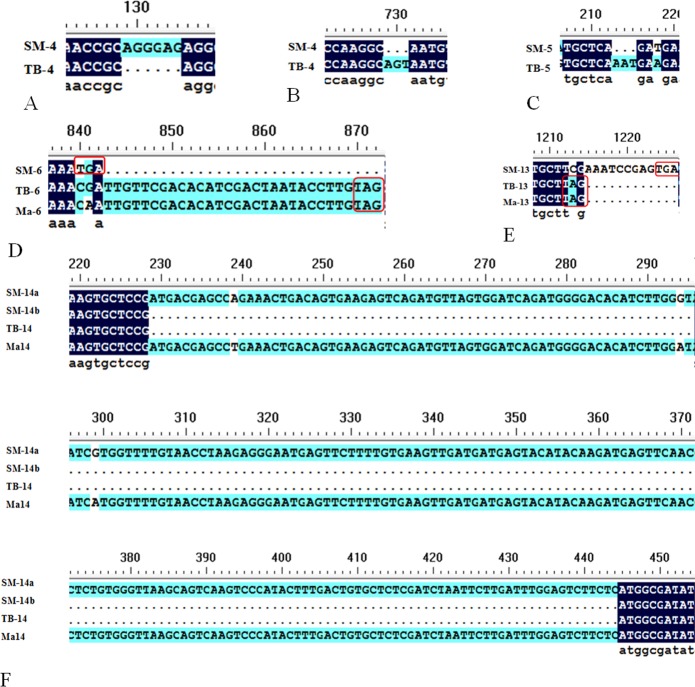
Alignment of part cDNA sequence of *CKII* family members from A genome, the wild banana and ‘Tianbaojiao’. Alignment analyses of the *CKII* family genes were performed using DNAMAN. A-C, Alignment of *CKIIβ-4-2* and *CKIIβ-4-3* from the wild banana and ‘Tianbaojiao’, showing the sequence differences of *CKIIβ-4-2* and *CKIIβ-4-3* between the wild banana and ‘Tianbaojiao’; D-E, Alignment of *CKIIβ-3-like* and *CKIIα-4* from the A genome, wild banana and ‘Tianbaojiao’, showing the sequence differences of *CKIIβ-3-like* and *CKIIα-4* with corresponding translational termination codon in this three *Musa* plants; F, Alignment of *CKIIβ-like-2* from the A genome, wild banana and ‘Tianbaojiao’, showing the 216 bp sequence deletion of *CKIIβ-like-2b* from the wild banana and *CKIIβ-like-2* from ‘Tianbaojiao’. A genome, the wild banana and ‘Tianbaojiao’ denoted as Ma, SM and TB. The *CKII* family members of *CKIIβ-4-2*, *CKIIβ-4-3*, *CKIIβ-3-like*, *CKIIα-4*, *CKIIβ-like-2a*, and *CKIIβ-like-2b*, were abbreviated 4, 5, 6, 13, 14a, and 14b.

Using RT-PCR, 13 cDNA sequences of the *CKII* family members were obtained from the *Musa* spp. cv. ‘Tianbaojiao’ and wild banana (*Musa itinerans*), respectively (Table 1). The sequence lengths of 9 members were the same but those of the other 4 members were different between the ‘Tianbaojiao’ and the wild banana (Table 1). Between them, both *CKIIβ-4-2* and *CKIIβ-4-3* had sequences that differed by 3–6 bp (Fig 1A, Fig 1B and 1C), and *CKIIβ-3-like* (Fig 1D) and *CKIIα-4* (Fig 1E) also had sequence differences, making them more similar to those of genome A, which resulted in different stop codons. In addition, there were 2 transcripts of *CKIIβ-like-2* in wild banana, and one (*MiCKIIβ-like-2a*) was similar to that of genome A, while the other (*MiCKIIβ-like-2b*) was similar to that of the ‘Tianbaojiao’ banana (*MaCKIIβ-like-2*) (Fig 1F). The sequence analysis indicated that the *MiCKIIβ-like-2a* and *MiCKIIβ-like-2b* genes had a 216 bp sequence difference, which belonged to an exon region when compared with the A genome. Therefore, we inferred that an alternative splicing event during evolution resulted in an exon deletion in the transcripts of *MiCKIIβ-like-2b* and *MaCKIIβ-like-2*. The sequence comparisons of *CKII* family members among wild banana, ‘Tianbaojiao’ and genome A were conducted (Table 2). Most of the members were the same or similar among these comparisons, except for the alternatively spliced transcripts of *MiCKIIβ-like-2b* and *MaCKIIβ-like-2*, which were both quite different from the transcripts of the genome A. In addition, the *CKII* α subunit members were generally similar, while *CKII* β subunit members were relatively different.

**Table 1:** The nucleotide sequences characteristics of *CKII* family members between the wild banana and ‘Tianbaojiao’.

*CKII* family in wild banana			*CKII* family in ‘Tianbaojiao’		
Gene name	ORF (bp)	Accession NO.	Gene name	ORF (bp)	Accession NO.
*MiCKIIα-1*	1002	MF598885	*MaCKIIα-1*	1002	MF598873
*MiCKIIβ-4-1*	843	MF598886	*MaCKIIβ-4-1*	843	MF598874
*MiCKIIβ-like-1*	681	MF598887	*MaCKIIβ-like-1*	681	MF598875
*MiCKIIβ-4-2*	861	MF598888	*MaCKIIβ-4-2*	858	MF598876
*MiCKIIβ-4-3*	840	MF598889	*MaCKIIβ-4-3*	843	MF598877
*MiCKIIβ-3-like*	843	MF598890	*MaCKIIβ-3-like*	873	MF598878
*MiCKIIβ-4-4*	843	MF598891	*MaCKIIβ-4-4*	843	MF598879
*MiCKIIα-2*	1251	MG451828	*MaCKIIα-2*	1251	MF598880
*MiCKIIα-3*	1002	MF598892	*MaCKIIα-3*	1002	MF598881
*MiCKIIα-4*	1227	MF598893	*MaCKIIα-4*	1215	MG451818
*MiCKIIβ-like-2a*	855	MF598894	*MaCKIIβ-like-2*	639	MF598882
*MiCKIIβ-like-2b*	639	MF598895			
*MiCKIIα-5*	1176	MF598896	*MaCKIIα-5*	1176	MF598883
*MiCKIIβ-like-3*	843	MF598897	*MaCKIIβ-like-3*	843	MF598884

**Table 2:** The sequences comparisons of the *CKII* family members among the wild banana, ‘Tianbaojiao’ and the A genome.

The wild banana			‘Tianbaojiao’			Genome A		
Gene name	Consistency (%)		Gene name	Consistency (%)		Gene ID	Consistency (%)	
*MiCKIIα-1*	91.82	79.42	*MaCKIIα-1*	93.71	79.88	Ma06_p18320.2	92.61	79.54
*MiCKIIα-3*			*MaCKIIα-3*			Ma10_p11700.1		
*MiCKIIα-5*			*MaCKIIα-5*			Ma05_p01580.1		
*MiCKIIα-2*	84.68		*MaCKIIα-2*	85.37		Ma09_p09220.1	86.01	
*MiCKIIα-4*			*MaCKIIα-4*			Ma06_p36630.1		
*MiCKIIβ-4-1*	96.96	91.55	*MaCKIIβ-4-1*	96.28	91.43	Ma04_p36590.1	94.78	90.48
*MiCKIIβ-4-3*			*MaCKIIβ-4-3*			Ma02_p12220.2		
*MiCKIIβ-4-4*			*MaCKIIβ-4-4*			Ma04_p32940.1		
*MiCKIIβ-4-2*	81.71		*MaCKIIβ-4-2*	82.11		Ma04_p18400.1	81.13	
*MiCKIIβ-like-3*		59.55	*MaCKIIβ-like-3*		59.67	Ma05_p16000.1		80.30
*MiCKIIβ-like-2b*	73.57		*MaCKIIβ-like-2*			Ma08_p15420.1		
*MiCKIIβ-like-2a*								
*MiCKIIβ-like-1*	68.56		*MaCKIIβ-like-1*	66.90		Ma02_p23160.1	67.12	
*MiCKIIβ-3-like*			*MaCKIIβ-3-like*			Ma06_p13370.1		

The analysis of the functional domains indicated that all of the CKII family members contained the whole STKc_CK2_alpha or CK_II_beta conserved domains in wild banana and ‘Tianbaojiao’.

### 3.3 Cloning and analyses of the *CKIIβ-like-2* gDNA sequences in wild banana and ‘Tianbaojiao’

The gDNA sequences of *CKIIβ-like-2* in wild banana and ‘Tianbaojiao’ was cloned, and the gene structures of *MiCKIIβ-like-2a*, *MiCKIIβ-like-2b* and *MaCKIIβ-like-2* were predicted to further validate the alternative splicing of *CKIIβ-like-2* in wild banana and ‘Tianbaojiao’ (S5 Fig). The results showed that, 4176 bp and 4164 bp *CKIIβ-like-2* gDNA sequences in wild banana and ‘Tianbaojiao’ were obtained, respectively. And the gene structures analysis showed 5 exons and 4 introns existed in *MiCKIIβ-like-2a*, while there was one deletion exons in *MiCKIIβ-like-2b* in wild banana. The *MaCKIIβ-like-2* in ‘Tianbaojiao’ also has 4 exons and 3 introns, similar with *MiCKIIβ-like-2b* in wild banana. So, the *MiCKIIβ-like-2b* in wild banana and *MaCKIIβ-like-2* in ‘Tianbaojiao’ might be the exon deletion alternative splicing transcript.

### 3.4 Predicted subcellular localizations of the CKII family members among wild banana, ‘Tianbaojiao’ and the A genome

The subcellular localization of the CKII family members among wild banana, ‘Tianbaojiao’ and the A genome were predicted (Table 3).

**Table 3:** Predicted and analysis of the subcellular localization of the CKII family members among the wild banana, ‘Tianbaojiao’ and A genome.

Subunit	Genome A		The wild banana		‘Tianbaojiao’	
	Gene ID	subcellular localization	Gene name	subcellular localization	Gene name	subcellular localization
**α**	Ma06_p18320.1	cytoplasmic	MiCKIIα-1	cytoplasmic	MaCKIIα-1	cytoplasmic
**β**	Ma04_p36590.1	nuclear	MiCKIIβ-4-1	nuclear	MaCKIIβ-4-1	nuclear
**β**	Ma02_p23160.1	cytoplasmic	MiCKIIβ-like-1	nuclear	MaCKIIβ-like-1	cytoplasmic
**β**	Ma04_p18400.1	nuclear	MiCKIIβ-4-2	nuclear	MaCKIIβ-4-2	nuclear
**β**	Ma02_p12220.1	nuclear	MiCKIIβ-4-3	nuclear	MaCKIIβ-4-3	nuclear
**β**	Ma06_p13370.1	nuclear	MiCKIIβ-3-like	nuclear	MaCKIIβ-3-like	nuclear
**β**	Ma04_p32940.1	nuclear	MiCKIIβ-4-4	nuclear	MaCKIIβ-4-4	nuclear
**α**	Ma09_p09220.1	mitochondrial	MiCKIIα-2	mitochondrial	MaCKIIα-2	mitochondrial
**α**	Ma10_p11700.1	cytoplasmic	MiCKIIα-3	cytoplasmic	MaCKIIα-3	cytoplasmic
**α**	Ma06_p36630.1	mitochondrial	MiCKIIα-4	mitochondrial	MaCKIIα-4	mitochondrial
**β**	Ma08_p15420.1	nuclear	MiCKIIβ-like-2a	nuclear	MaCKIIβ-like-2	nuclear
			MiCKIIβ-like-2b	nuclear		
**α**	Ma05_p01580.1	extracellula, including cell wall	MiCKIIα-5	extracellula, including cell wall	MaCKIIα-5	extracellular, including cell wall
**β**	Ma05_p16000.1	nuclear	MiCKIIβ-like-3	nuclear	MaCKIIβ-like-3	nuclear

Comparisons of the subcellular localizations among the CKII family indicated that all of the members, except for MiCKIIβ-like-1 from wild banana, were the same among wild banana, ‘Tianbaojiao’ and the A genome. Furthermore, all of the CKII β subunit members, except for CKIIβ-like-1 from ‘Tianbaojiao’ and the A genome, were localized to the nucleus. However, the subcellular localizations of the CKII α subunit members varied, including cytoplasmic, mitochondrial, and extracellular (including cell wall). In particularly, MaCKIIα-4, Ma06_p36630.1 and MiCKIIα-4 were predicted to localize to the mitochondrial with 100%, 100%, and 95.7% probabilities, respectively, which indicated that CKIIα-4 likely functioned in the mitochondria.

**Fig 2:**
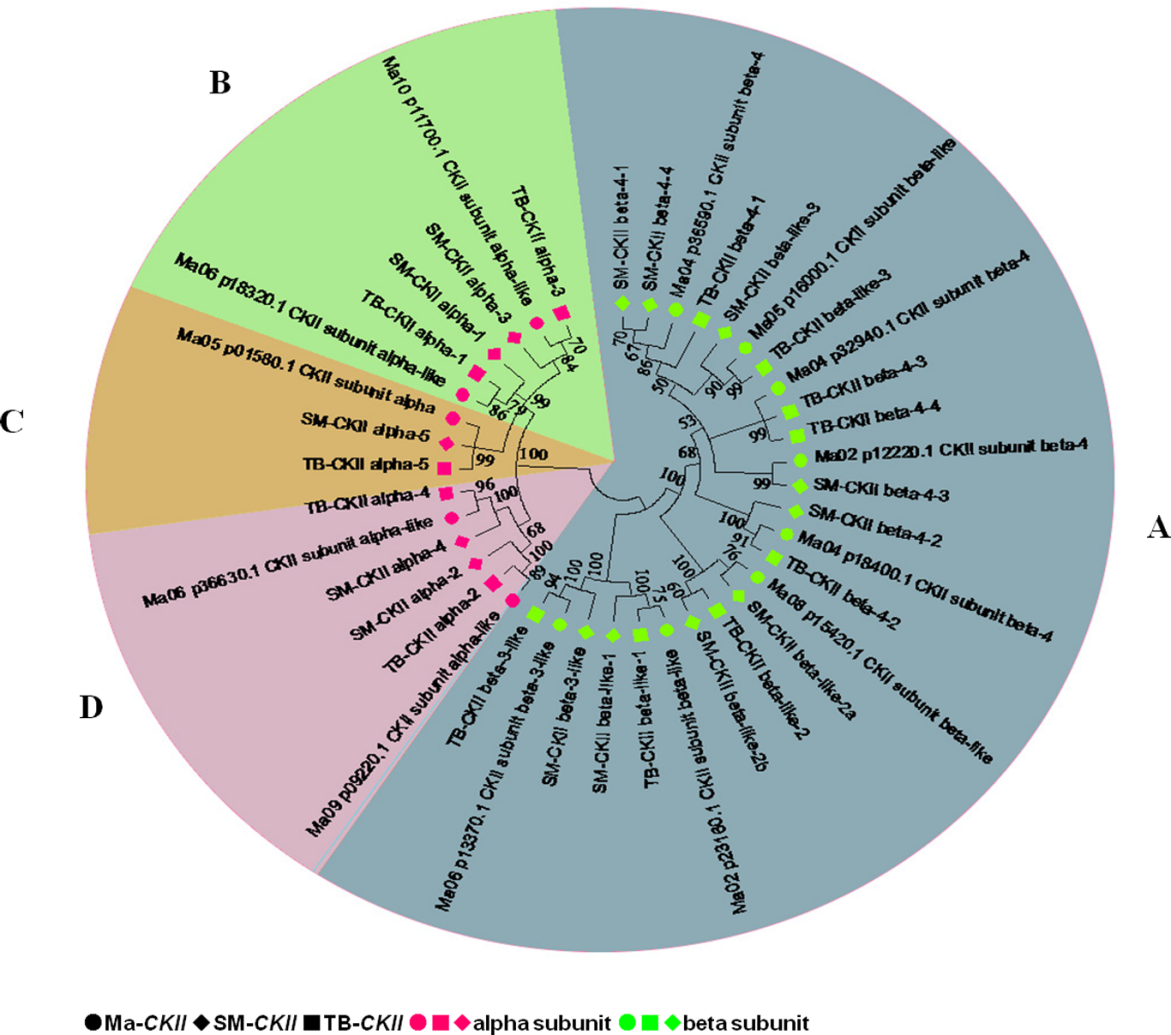
Phylogenetic analysis of the CKII proteins in A genome, the wild banana and ‘Tianbaojiao’. The phylogenetic tree was constructed using MEGA 6.0 by the neighbor-joining (NJ) method and 1000 bootstrap replicates. The tree was divided into four phylogenetic subgroups, designated A, B, C and D. The CKII β members were assigned to one branch (A), which were localized to the nucleus except for CKIIβ-like-1 from ‘Tianbaojiao’ and the A genome. The CKII α members were assigned to another branch, and being further clustered, which were consistent with subcellular localizations site, i.e. cytoplasmic (B), extracellular, including cell wall (C) and mitochondrial (D). A genome, the wild banana and ‘Tianbaojiao’ denoted as Ma, SM and TB.

The clustering analysis, combined with the subcellular localizations of the CKII family members from the wild banana, ‘Tianbaojiao’ and the A genome are shown in Fig 2, and both the CKII α and the CKII β members were clearly assigned to two branches. The CKII β members were assigned to one branch (Fig 2A), which were localized to the nucleus except for CKIIβ-like-1 from ‘Tianbaojiao’ and the A genome. The CKII α members were assigned to another branch, and being further clustered which were consistent with subcellular localizations site, i.e. cytoplasmic (Fig 2B), extracellular, including cell wall (Fig 2C) and mitochondrial (Fig 2D).

### 3.5 Analyses *CKII* family members gene structures among the wild banana, ‘Tianbaojiao’ and A genome

**Fig 3:**
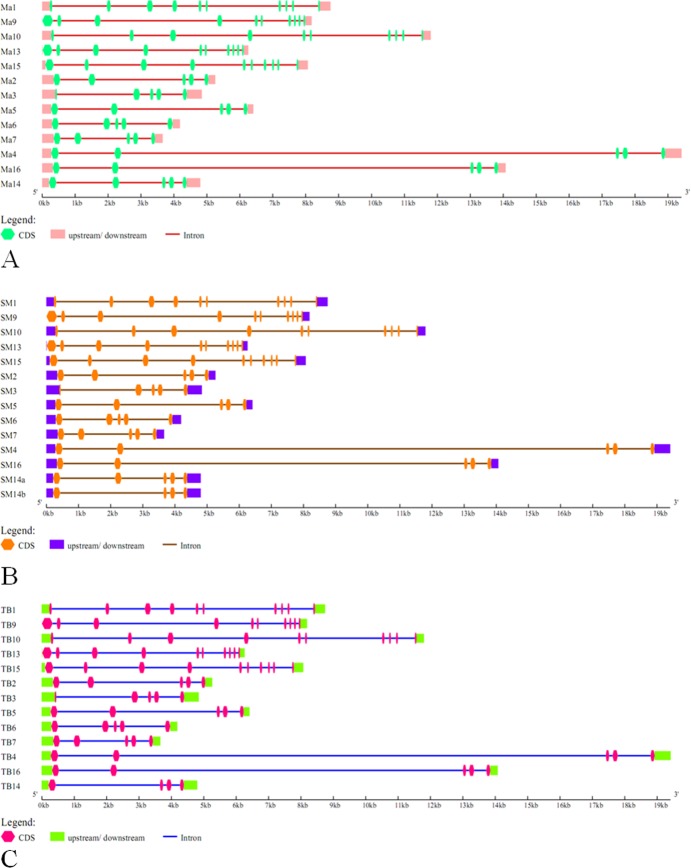
Gene structure of *CKII* family members in A genome, the wild banana and ‘Tianbaojiao’. Structural analyses of the *CKII* family genes were performed using GSDS. The exons (CDS) and introns are represented by colored boxes and lines, respectively. A, the gene structure of *CKII* family members from A genome; B, the gene structure of *CKII* family members from the wild banana; C, the gene structure of *CKII* family members from ‘Tianbaojiao’. A genome, the wild banana and ‘Tianbaojiao’ denoted as Ma, SM and TB. The *CKII* gene family members of *CKIIα-1*, *CKIIβ-4-1*, *CKIIβ-like-1*, *CKIIβ-4-2*, *CKIIβ-4-3*, *CKIIβ-3-like*, *CKIIβ-4-4*, *CKIIα-2*, *CKIIα-3*, *CKIIα-4*, *CKIIβ-like-2a*, *CKIIβ-like-2b*, *CKIIα-5*, *CKIIβ-like-3*, were abbreviated 1, 2, 3, 4, 5, 6, 7, 9, 10, 13, 14a, 14b, 15 and 16.

The gene structures of the *CKII* family members among wild banana, ‘Tianbaojiao’ and the A genome were analyzed (Fig 3). The *CKII* α members contained 10 exons and 9 introns, while all the *CKII* β members contained 5 exons and 4 introns, except for *MiCKIIβ-like-2b* from the wild banana and *MaCKIIβ-like-2* from ‘Tianbaojiao’, which contained 4 exons and 3 introns. The differences in these two members may have resulted from an alternative splicing event during evolution that resulted in an exon deletion. The structures define the functions, therefore, the *CKII* α and β subunit members likely functioned differently.

### 3.6 Predicted occurrence of CKII phosphorylation sites among the wild banana, ‘Tianbaojiao’ and A genome

CKII phosphorylation sites were predicted among the wild banana, ‘Tianbaojiao’ and A genome (S3 Table). The results showed that the numbers of phosphorylation sites in the CKII family members among the wild banana, ‘Tianbaojiao’ and A genome varied from 1 to 12, and the numbers of phosphorylation sites in the CKII β members (except for the proteins from the 2 alternatively spliced transcripts MiCKIIβ-like-2b and MaCKIIβ-like-2) were generally greater than those in the CKII α members. The former were between 5 and 12, and the latter were between 1 and 2, which was in accordance with the differences in the functions of CKII α and CKII β. The CKII α members acted mainly as catalysts of phosphorylation, while the CKII β members were highly conserved and acted mainly in the regulation of enzymatic activities.

The sites and numbers of amino acid residues of CKII phosphorylation in CKIIα-1, CKIIβ-4-4, CKIIα-2, CKIIα-3 and CKIIα-4 members were the same, while the others showed some differences among the wild banana, ‘Tianbaojiao’ and A genome. For example, the amino acid residue serine 104 in Ma05_p01580.1 and serine 148 in Ma04_p36590.1 were both specific to the A genome, while the amino acid residue serine 10 in MiCKII β-4-3 was specific to wild banana.

### 3.7 Analysis of the conserved motifs of CKII family among the wild banana, ‘Tianbaojiao’ and A genome

**Fig 4:**
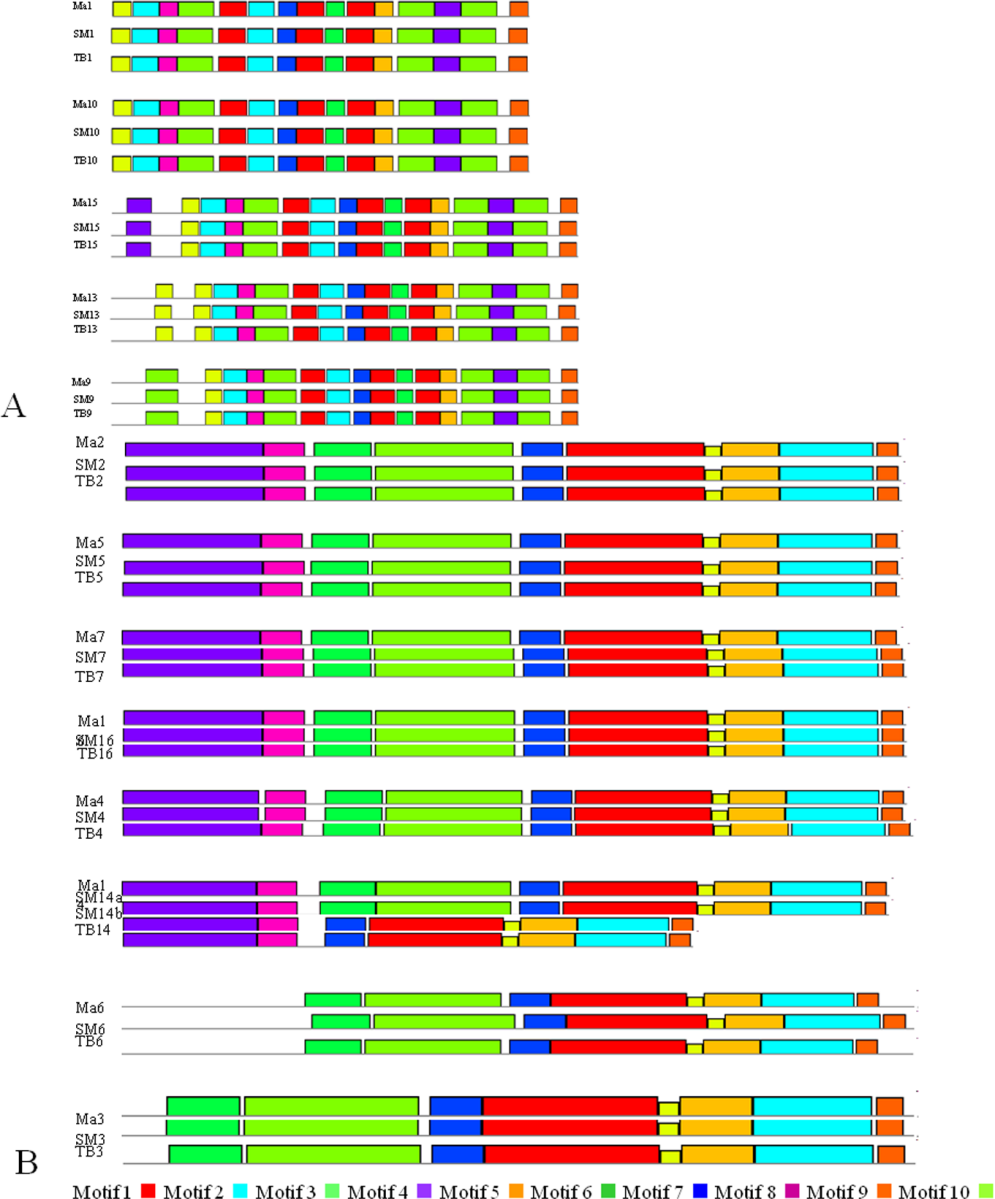
Conserved motifs analysis of CKII family members proteins in A genome, the wild banana and ‘Tianbaojiao’ showing the different conserved motifs of α subunits and β subunits members of CKII. The conserved motifs of the CKII proteins were identified using Multiple Em for Motif Elicitation (MEME). In total, 10 motifs were identified of α subunits (A) and β subunits (B) members of CKII, respectively and are shown in different colors. A genome, the wild banana and ‘Tianbaojiao’ denoted as Ma, SM and TB. The CKII gene family members of CKIIα-1, CKIIβ-4-1, CKIIβ-like-1, CKIIβ-4-2, CKIIβ-4-3, CKIIβ-3-like, CKIIβ-4-4, CKIIα-2, CKIIα-3, CKIIα-4, CKIIβ-like-2a, CKIIβ-like-2b, CKIIα-5, CKIIβ-like-3, were abbreviated 1, 2, 3, 4, 5, 6, 7, 9, 10, 13, 14a, 14b, 15 and 16.

The conserved motifs of CKII family members were analyzed among the wild banana, ‘Tianbaojiao’ and A genome (Fig 4). All 5 CKII α members had the same numbers of the 10 conserved motifs in the wild banana, ‘Tianbaojiao’ and A genome. However, CKII α-2 had the specific motif 3, CKII α-4 had the specific motif 10 and CKII α-5 had the specific motif 4 (Fig 4A). The CKII β members of CKII β-4-1, CKII β-4-2, CKII β-4-3, CKII β-4-4 and CKII β-like-3 in both A genome and the wild banana, CKII β-like-2 in A genome and CKII β-like-2a in wild banana, had the same numbers of the 10 conserved motifs. However, CKII β-like-2b and CKII β-like-2 both had 8 conserved motifs, lacking motifs 3 and 6, and CKII β-like-1 and CKII β-3-like both had 8 conserved motifs, lacking motifs 4 and 8 (Fig 4B). Thus, the motifs among CKII α or CKII β family members were highly conserved.

### 3.8 Expression levels of the *CKII* family members in wild banana at different temperatures and in different tissues by qPCR

**Fig 5:**
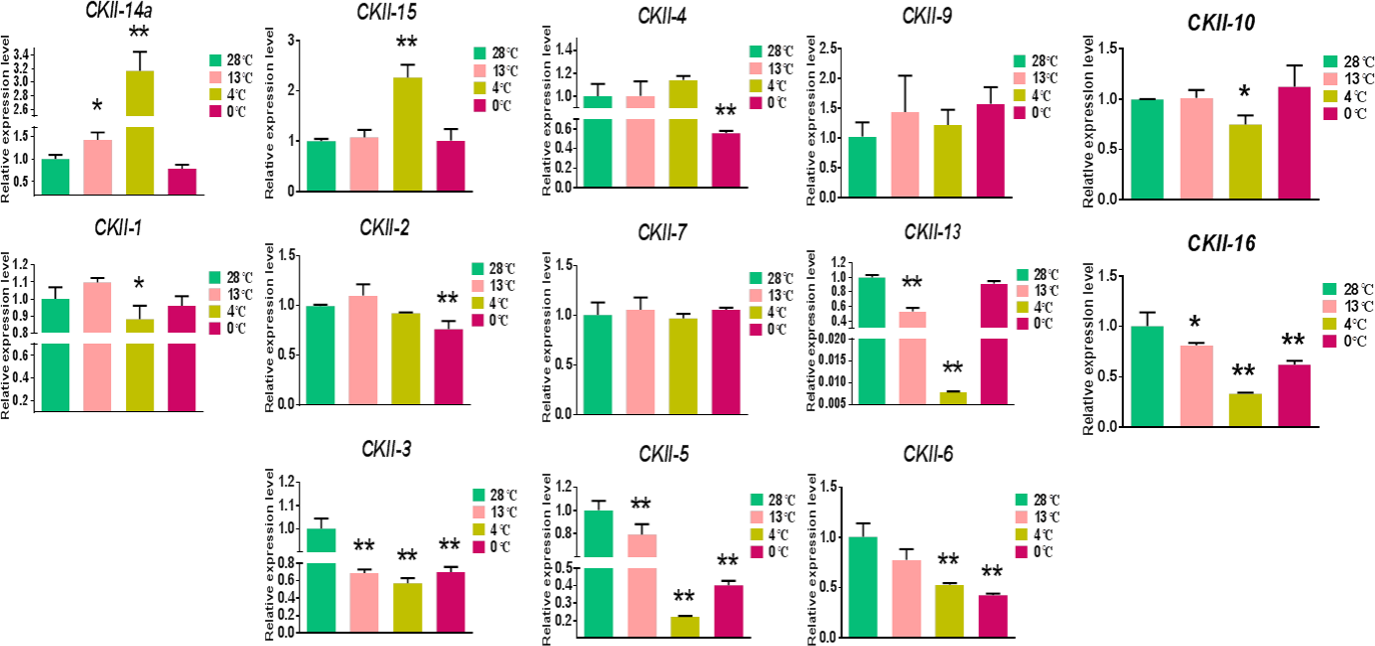
Gene expression of *CKII* family members at different temperatures in the wild banana. Transcripts abundance was quantified using qRT-PCR. SPSS software was used to perform all statistical analyses of data, and all data are expressed as the means ± SDs of three independent replicates. Duncan’s multiple range test was used for the significant differences. *: significant difference (at p-value <0.05) identified by comparing with 28°C, **: very significant difference (at p-value <0.01) identified by comparing with 28°C. The *CKII* gene family members of *CKIIα-1*, *CKIIβ-4-1*, *CKIIβ-like-1*, *CKIIβ-4-2*, *CKIIβ-4-3*, *CKIIβ-3-like*, *CKIIβ-4-4*, *CKIIα-2*, *CKIIα-3*, *CKIIα-4*, *CKIIβ-like-2a*, *CKIIα-5*, *CKIIβ-like-3*, were abbreviated *CKII-1*, *CKII-2*, *CKII-3*, *CKII-4*, *CKII-5*, *CKII-6*, *CKII-7*, *CKII-9*, *CKII-10*, *CKII-13*, *CKII-14a*, *CKII-15* and *CKII-16*.

**Fig 6:**
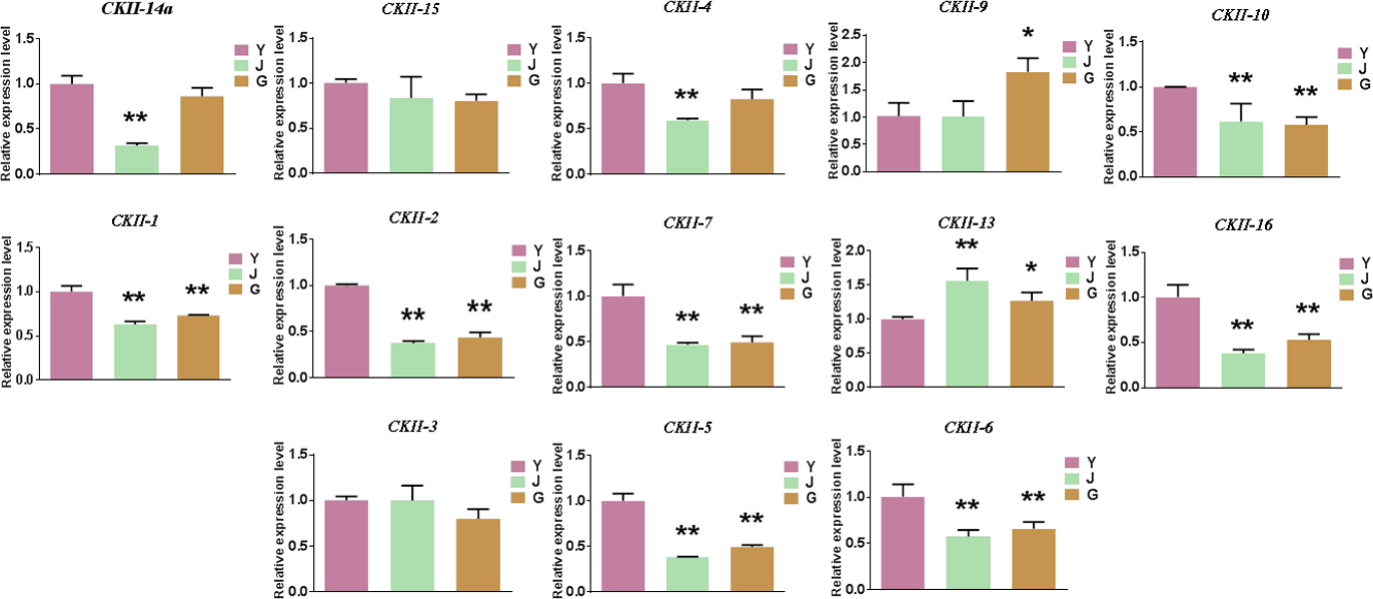
Gene expression of *CKII* family members in different tissues in the wild banana. Transcripts abundance was quantified using qRT-PCR. SPSS software was used to perform all statistical analyses of data, and all data are expressed as the means ± SDs of three independent replicates. Duncan’s multiple range test was used for the significant differences. *: significant difference (at p-value <0.05) identified by comparing with 28°C, **: very significant difference (at p-value <0.01) identified by comparing with 28°C. G, roots; J, pseudo-stems; Y, leaves. The *CKII* gene family members of *CKIIα-1*, *CKIIβ-4-1*, *CKIIβ-like-1*, *CKIIβ-4-2*, *CKIIβ-4-3*, *CKIIβ-3-like*, *CKIIβ-4-4*, *CKIIα-2*, *CKIIα-3*, *CKIIα-4*, *CKIIβ-like-2a*, *CKIIα-5*, *CKIIβ-like-3*, were abbreviated *CKII-1*, *CKII-2*, *CKII-3*, *CKII-4*, *CKII-5*, *CKII-6*, *CKII-7*, *CKII-9*, *CKII-10*, *CKII-13*, *CKII-14a*, *CKII-15* and *CKII-16*.

The expression levels of the *CKII* family members in wild banana were detected by qPCR in leaf tissue at different temperatures (growth temperature of 28°C as the control, and the low temperatures of 13°C for critical growth, 4°C for chilling and 0°C for freezing) (Fig 5) and in different tissues (leaves, pseudo-stems and roots) (Fig 6). The expression levels of *CKIIβ-like-2a*, *CKIIα-5* and *CKIIβ-4-2* were the highest at 4°C, especially those of *CKIIβ-like-2a* and *CKIIα-5*, which were significantly higher than those of the control, and the expression levels of *CKIIβ-like-2a*, *CKIIα-5* and *CKIIβ-4-2* in leaves were also higher than those in pseudo-stems and roots. The expression level of *CKIIα-3* was lowest at 4°C, which was highest in leaf. While the expression level of *CKIIα-2* in root was higher than that in leaf (the only member). The expression levels of *CKIIα-1*, *CKIIβ-4-1* and *CKIIβ-4-4* were all high, but not significant, at 13°C. The expression levels of these 3 members in leaves were significantly higher than in the other two tissues, even though they showed similar expression patterns. The expression levels of the 5 members, *CKIIβ-like-1*, *CKIIβ-4-3*, *CKIIβ-3-like*, *CKIIα-4* and *CKIIβ-like-3*, changed remarkably at 2 or 3 of the low temperature points, and the expression patterns of *CKIIβ-like-1*, *CKIIβ-4-3*, *CKIIα-4* and *CKIIβ-like-3* were similar at 28°C, 13°C and 4°C. Interestingly, the expression level of *CKIIα-4* at 0°C was 117 times higher than at 4°C, and it had a significantly higher expression level in pseudo-stem than that in leaves. It was also the only member that expressed more highly in pseudo-stem than in leaves and roots. *CKIIα-2* and *CKIIα-4*, with root and pseudo-stem specific expression, both belonged to *CKII* α subunit members and had a sequence similarity of 84.68%, and were expressed relatively higher at 0°C. This suggested that wild banana could activate not only the *CKII* members in leaves but also the *CKII* members in roots and pseudo-stems for cold acclimation in response to the 0°C cold stress. In addition, alternatively spliced transcript *CKIIβ-like-2b* was expressed at very low levels or could not be detected at different temperatures and in different tissues (data not shown), which was quite different from the expression patterns of the normal transcript *CKIIβ-like-2a*. The wild banana of *Musa itinerans* from Sanming City and A genome from ‘DH-Pahang’ (*Musa accuminata*, AA group) of the Malaysian wild banana had the normal transcript *CKIIβ-like-2a*, but the cultivated ‘Tianbaojiao’ (AAA group) had only the lowly expressed alternatively spliced transcript *CKIIβ-like-2*. It might be a key gene related to cold stress. To further validate the expression levels of alternatively spliced transcript *CKIIβ-like-2* in response to different temperatures in cultivated banana. For above mentioned reasons that the ‘Tianbaojiao’ cannot grow well at 4°C low temperature stress, the expression profile of alternatively spliced transcript *CKIIβ-like-2* was performed at 28°C (control) and 13°C in ‘Tianbaojiao’ (S6 Fig). The expression level of *CKIIβ-like-2* at 13°C was lower than those of the control. The growth of ‘Tianbaojiao’ is retarded or stopped at 13°C, which is thought to be the critical temperature of growth and relatively low temperature for most banana cultivars in China, while *CKIIβ-like-2* was down-regulated expression at 13°C in ‘Tianbaojiao’. So the alternatively spliced transcript *CKIIβ-like-2* respond negatively to 13°C low temperature in ‘Tianbaojiao’, which is opposite trend for the normal transcript *CKIIβ-like-2a* at 13°C low temperature in wild banana.

## Discussion

### 4.1 The function of *CKII* family members might be different in *Musa* plants

*CKII* is considered a tetrameric complex consisting of two catalytic α subunits and two regulatory β subunits [14, 29–30]. The gene structures, phosphorylation sites, and conserved motifs of CKII α and β members were specific in three *Musa* plants. In addition, most of the CKII β subunit members localized in the nucleus, but the CKII α subunit members varied in *Musa* plants. However, it is opposite in *Arabidopsis*. All of the CKII α members, except for the members of Alphacp (localized in chloroplast), localized to the nucleus, but the CKII β members localized to various sites in *Arabidopsis* [14]. CKII subunits localized frequently to the nucleus and cytosol, but they have also been found in other organelles, such as mitochondria, the endoplasmic reticulum and the external and internal surfaces of the plasma membrane [14, 31–32]. In plants, CKII has been found to localize to the cytosol and the nucleus [16], as well as to the chloroplast in mustard and *Arabidopsis* [8, 14]. In this study, the members of CKII family localized mostly in the nucleus, followed by the cytoplasmic and mitochondrial, and then extracellular sites. The maize *CKII* α subunit was the first catalytic subunit identified in plants [33], and contains 10 exons separated by 9 introns. Similarly, the *CKII* α members contained 10 exons and 9 introns in three *Musa* plants. The characteristic of *CKII* α subunits are highly conserved among different species were reported [10]. In present study, all the *CKII* β members contained 5 exons and 4 introns except for the alternatively spliced transcript. All the plant CKII β proteins present the major conserved features described for CKII β subunits from other organisms [34]. This high-degree conservation indicates that the *CKII* functions may be conserved between the different species. In addition, the conserved motifs analysis of CKII family members in three *Musa* plants showed the motifs among CKII α or CKII β family members were highly conserved. These above results may further illustrate protein kinase *CKII* is a ubiquitous and highly conserved Ser/Thr kinase. The numbers of CKII phosphorylation sites in the CKII β members (except for the 2 alternatively spliced transcripts) were generally greater than those in the CKII α members in three *Musa* plants, and it will be worth investigating relevance of *CKII* subunit functions.

Expression levels of *CKII* family members at different temperatures and in different tissues were also different in wild banana. In particularly, the wild banana of *Musa itinerans* contained both the normal *CKIIβ-like-2a* transcript and the alternatively spliced *CKIIβ-like-2b* transcript. Genome A had *CKIIβ-like-2a*, but the cultivated ‘Tianbaojiao’ had only alternatively spliced transcript *CKIIβ-like-2*. The two transcripts of *CKIIβ-like-2* had distinct expression levels in response to low temperature. The three *Musa* plants differ in their responses to environmental stress [20, 35–39], and *CKII* is involved in various plant developmental processes and in responses to biotic and abiotic stresses [2–3, 40]. Plant breeders require access to genetic diversity to satisfy the demands for more and higher quality foods that can be produced in a variable or changing climate, and the crop’ wild relatives represent a practical gene pool from which to expand the genetic diversity in crop plants [41]. Ortiza and Swennenb (2014) [42] proposed changing from crossbreeding to the biotechnology-facilitated improvement of banana and plantain. *CKIIβ-like-2a* may be a target gene for cold resistant in cultivated banana breeding by biotechnology technology.

### 4.2 The alternative splicing events of *CKII* may result from the complex origin and evolution of *Musa* lineage

The origin of *Musa* plant is quite complex. *Musa acuminata* (A genome) and *Musa balbisiana* (B genome) were the ancestors of *Musa* lineage, and the banana cultivars mainly involve both and are sometimes diploid but generally triploid. The ‘Tianbaojiao’ (*Musa* spp., Cavendish, AAA group) which is the famous traditional cultivar in China, originated from the wild banana *Musa acuminate* (AA group). The wild banana from Sanming City is *Musa itinerans*, which is one wild banana different from the *Musa acuminata* and *Musa balbisiana*. In additions, the *Musa* lineage experienced long-term evolution. Lescot et al. have provided the evidence of a whole-genome duplications (WGDs) event in the *Musa* lineage for the first time [43]. D’Hont et al. have detected three rounds of WGDs (denoted as α, β and γ) in the *Musa* lineage; α and β events was dated at a similar period around 65 Myr (million years) ago, and γ event occurred around 100 Myr ago [23]. After WGD, most (65.4%) of the genes included in the *Musa* α/β ancestral blocks are singletons and only 10% are retained in four copies, in agreement with the loss of most gene-duplicated copies [44]. Genes are more prone to be co-retained or co-lost after WGD [45]. WGDs have played a major role in angiosperm genome evolution [46]. Alternative splicing, which is common in plants, is a rapidly evolving process after gene duplication [47–48]. Alternative splicing could affect gene regulation, gene function and cause functional divergence between duplicates [47, 49]. Moreover, alternative splicing plays a crucial role in defense response of plants [50]. Abiotic stresses are known to cause changes in alternative splicing patterns in plants [51–52]. Plants alter splicing patterns in response to temperature stress [53–56]. After gene and genome duplication, alternative splicing patterns have diverged considerably in an organ- or stress-specific manner during the evolutionary history of *Arabidopsis* lineage [49]. These above reports may explain the phenomenon of alternative splicing events occurred in *Musa* plants. In our study, the alternative splicing events resulted in an exon deletion in both *MiCKIIβ-like-2b* from the wild banana and *MaCKIIβ-like-2* from ‘Tianbaojiao’. Both the wild bananas of and genome A had the normal *CKIIβ-like-2* transcript, *CKIIβ-like-2a*, which was highly expressed under cold stress in the wild banana. However, the cultivar ‘Tianbaojiao’ did not contain *CKIIβ-like-2a*, and had only the alternatively spliced transcript *CKIIβ-like-2*, which was expressed at very low levels or could not be detected in response to cold stress in wild banana and respond negatively to 13°C low temperature in ‘Tianbaojiao’. Furthermore, from single gene duplication to WGD, gene duplication has occurred throughout eukaryotic evolution and contributed greatly to the duplicated genes [49]. The majority of duplicated genes are retained gain new functions and/or expression patterns (neofunctionalization) [49]. In maize leaf, 13% of homology gene pairs have undergone regulatory neofunctionalization [57]. WGD has formed novel functions genes and altered expression patterns [58]. This may be the factors of the different expression patterns in response to cold stress of the two *CKIIβ-like-2* transcripts in wild banana. The character of poorly conserved between duplicated genes by WGD was reported [47]. The two *CKIIβ-like-2* transcripts are also not conserved in three *Musa* plants. Besides, Feng et al. suggested the WGD, segmental duplication and complex transcriptional regulation contributed to the gene expansion and mRNA diversity of the *MaSODs* by the genome-wide identification of *SOD* gene family in ‘Tianbaojiao’ [25]. The alternative splicing events were occurred with high frequency in previous study related *Musa* plant [59–61]. Therefore, the likely factors of alternative splicing events occurred in present study are the complex origin and long-term evolution of *Musa* lineage.

## Conclusions

In this study, based on the banana genome database, the cloning, identification and characterization of the *CKII* family members in *Musa* spp. cv. Tianbaojiao (AAA group) and the wild banana (*Musa itinerans*) were reported. 13 cDNA sequences of the *CKII* family members were obtained from the ‘Tianbaojiao’ and the wild banana, respectively. The bioinformatics and qPCR analyses of *CKII* family members suggested that the function of *CKII* family members might be different in *Musa* plants. Furthermore, *CKII β-like-2a* might be a gene related to cold resistant. In addition, the *CKII β-like-2* gDNA sequences in wild banana and ‘Tianbaojiao’ were obtained, and the analysis of sequences and gene structures showed the *MiCKIIβ-like-2b* in wild banana and *MaCKIIβ-like-2* in ‘Tianbaojiao’ might be the exon deletion alternative splicing transcripts. The alternative splicing events of *CKII β-like-2* may result from the complex origin and evolution of *Musa* lineage.

## Supporting information

S1 Fig The leaves phenotypes of the wild banana and ‘Tianbaojiao’ in January 2016 showing the different response to the cold stress of these two *Musa* plants. A-B, ‘Tianbaojiao’ in the field; C, the wild banana in the field.
(TIF)

S2 Fig The leaves phenotypes of the wild banana and ‘Tianbaojiao’ at 4°C stress. A, ‘Tianbaojiao’ at 4°C, 3 h; B, ‘Tianbaojiao’ at 4°C, 5 h; C, ‘Tianbaojiao’ at 4°C, 7 h; D-E, 4°C stressed ‘Tianbaojiao’ at 28°C to recover; F, the wild banana at 4°C, 3 h; G, the wild banana at 4°C, 5 h; H, the wild banana at 4°C, 7 h.
(TIF)

S3 Fig The information of qPCR efficiency calibration curves for each primer pairs of *CKII* family genes and *CAC*. The amplification efficiency for each primer pairs of the *CKII* family genes and *CAC* was determined in a qPCR assay using a five-fold dilution series from a pooled cDNA template. A, *CKIIα-1*; B, *CKIIβ-4-1*; C, *CKIIβ-like-1*; D, *CKIIβ-4-2*; E, *CKIIβ-4-3*; F, *CKIIβ-3-like*; G, *CKIIβ-4-4*; H, *CKIIα-2*; I, *CKIIα-3*; J, *CKIIα-4*; K, *CKIIβ-like-2a*; L, *CKIIβ-like-2b*; M, *CKIIα-5*; N, *CKIIβ-like-3*; O, *CAC*.
(TIF)

S4 Fig **Phylogenetic analysis of the CKII family members in banana genome A and B.** The phylogenetic tree was constructed using MEGA5 by the neighbor-joining (NJ) method and 1000 bootstrap replicates. The tree was divided into two phylogenetic subgroups. The CKII β members of the two *Musa* plants were assigned to one branch, and the CKII α members combined with unclassified subunit CKII members were assigned to another branch.
(TIF)

S5 Fig Gene structures of *CKIIβ-like-2a*, *CKIIβ-like-2b* in wild banana and *CKIIβ-like-2* in ‘Tianbaojiao’. Structural analyses of *CKIIβ-like-2a*, *CKIIβ-like-2b* in wild banana and *CKIIβ-like-2* in ‘Tianbaojiao’ were performed using GSDS showing the *CKIIβ-like-2b* in wild banana and *CKIIβ-like-2* in ‘Tianbaojiao’ might be the exon deletion alternative splicing transcript. The exons and introns are represented by colored boxes and black lines, respectively. A, the gene structure of *MiCKIIβ-like-2a*; B, the gene structure of *MiCKIIβ-like-2b*; C, the gene structure of *MaCKIIβ-like-2*. The wild banana and ‘Tianbaojiao’ were abbreviated as ‘SM’ and ‘TB’. *CKIIβ-like-2* was abbreviated as ‘14’.
(TIF)

S6 Fig Gene expression of alternatively spliced transcript *CKIIβ-like-2* from ‘Tianbaojiao’ at 28°C and 13°C. Transcripts abundance was quantified using qRT-PCR. The expression levels from three independent biological replicates were analyzed.
(TIF)

S1 Table The primers used for gene cloning in this study. ‘Tianbaojiao’ and the wild banana were abbreviated as ‘TB’ and ‘SM’. The *CKII* gene family members of *CKIIα-1*, *CKIIβ-4-1*, *CKIIβ-like-1*, *CKIIβ-4-2*, *CKIIβ-4-3*, *CKIIβ-3-like*, *CKIIβ-4-4*, *CKIIα-2*, *CKIIα-3*, *CKIIα-4*, *CKIIβ-like-2a*, *CKIIβ-like-2b*, *CKIIα-5*, *CKIIβ-like-3*, were abbreviated *CKII-1*, *CKII-2*, *CKII-3*, *CKII-4*, *CKII-5*, *CKII-6*, *CKII-7*, *CKII-9*, *CKII-10*, *CKII-13*, *CKII-14a*, *CKII-14b*, *CKII-15* and *CKII-16*.
(DOC)

S2 Table The primers used for qPCR assay in this study. The *CKII* gene family members of *CKIIα-1*, *CKIIβ-4-1*, *CKIIβ-like-1*, *CKIIβ-4-2*, *CKIIβ-4-3*, *CKIIβ-3-like*, *CKIIβ-4-4*, *CKIIα-2*, *CKIIα-3*, *CKIIα-4*, *CKIIβ-like-2a*, *CKIIβ-like-2b*, *CKIIα-5*, *CKIIβ-like-3*, were abbreviated *CKII-1*, *CKII-2*, *CKII-3*, *CKII-4*, *CKII-5*, *CKII-6*, *CKII-7*, *CKII-9*, *CKII-10*, *CKII-13*, *CKII-14a*, *CKII-14b*, *CKII-15* and *CKII-16*.
(DOC)

S3 Table Predict of the CKII phosphorylation sites among the wild banana, ‘Tianbaojiao’ and genome A. ‘Ser’ and ‘Thr’ were abbreviated as ‘S’ and ‘T’.
(DOC)
