# Supplementary figures and images for: Genome-wide identification and characterization of the CKII gene family in the cultivated banana cultivar (Musa spp. cv Tianbaojiao) and the wild banana (Musa itinerans)
Source: PLoS One. 2018 Jul 11;13(7):e0200149. doi: 10.1371/journal.pone.0200149 (PMC6040749; doi:10.1371/journal.pone.0200149)

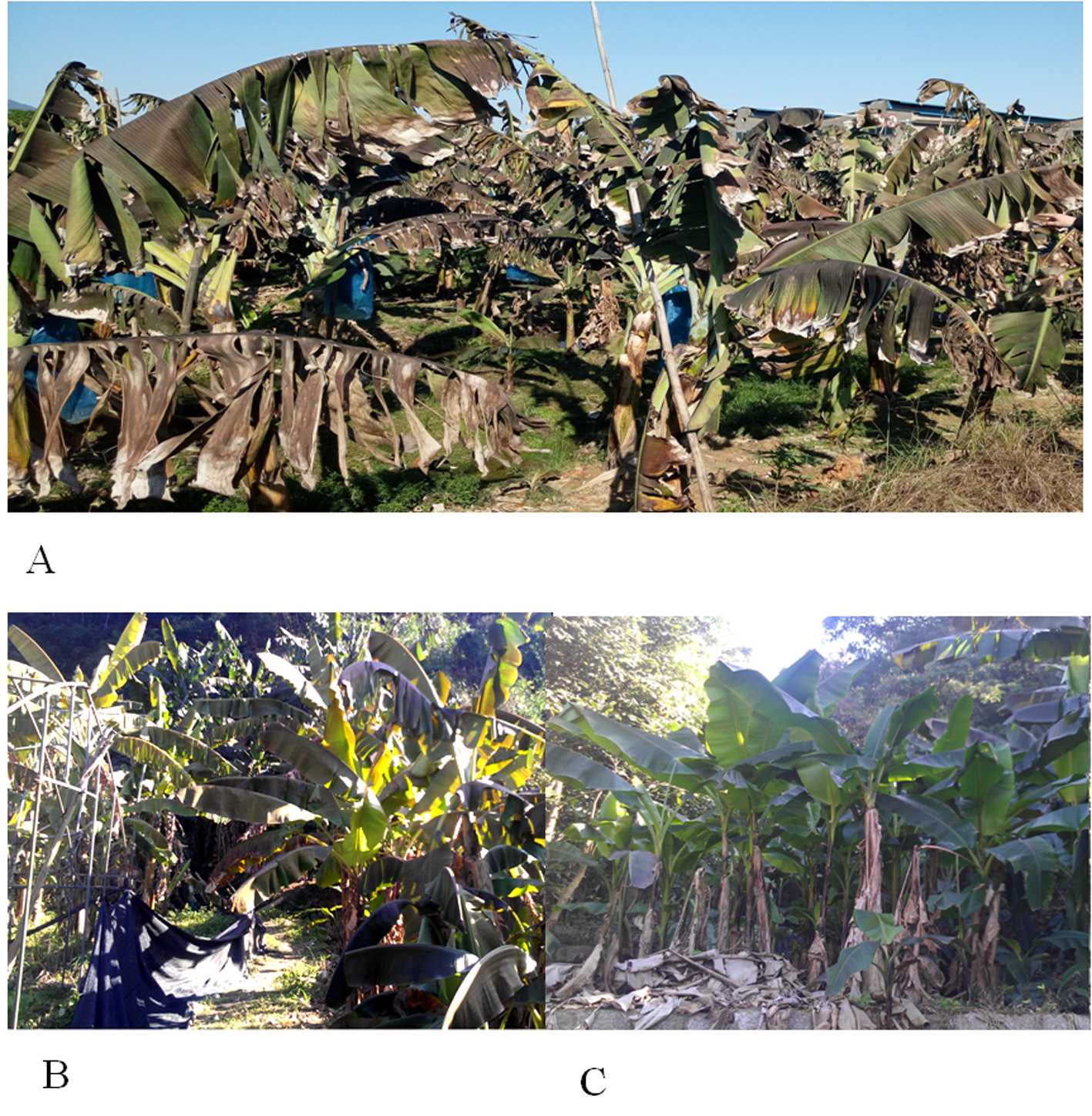

Supplement: S1 Fig — A-B, ‘Tianbaojiao’ in the field; C, the wild banana in the field. (TIF) [file pone.0200149.s001.tif]

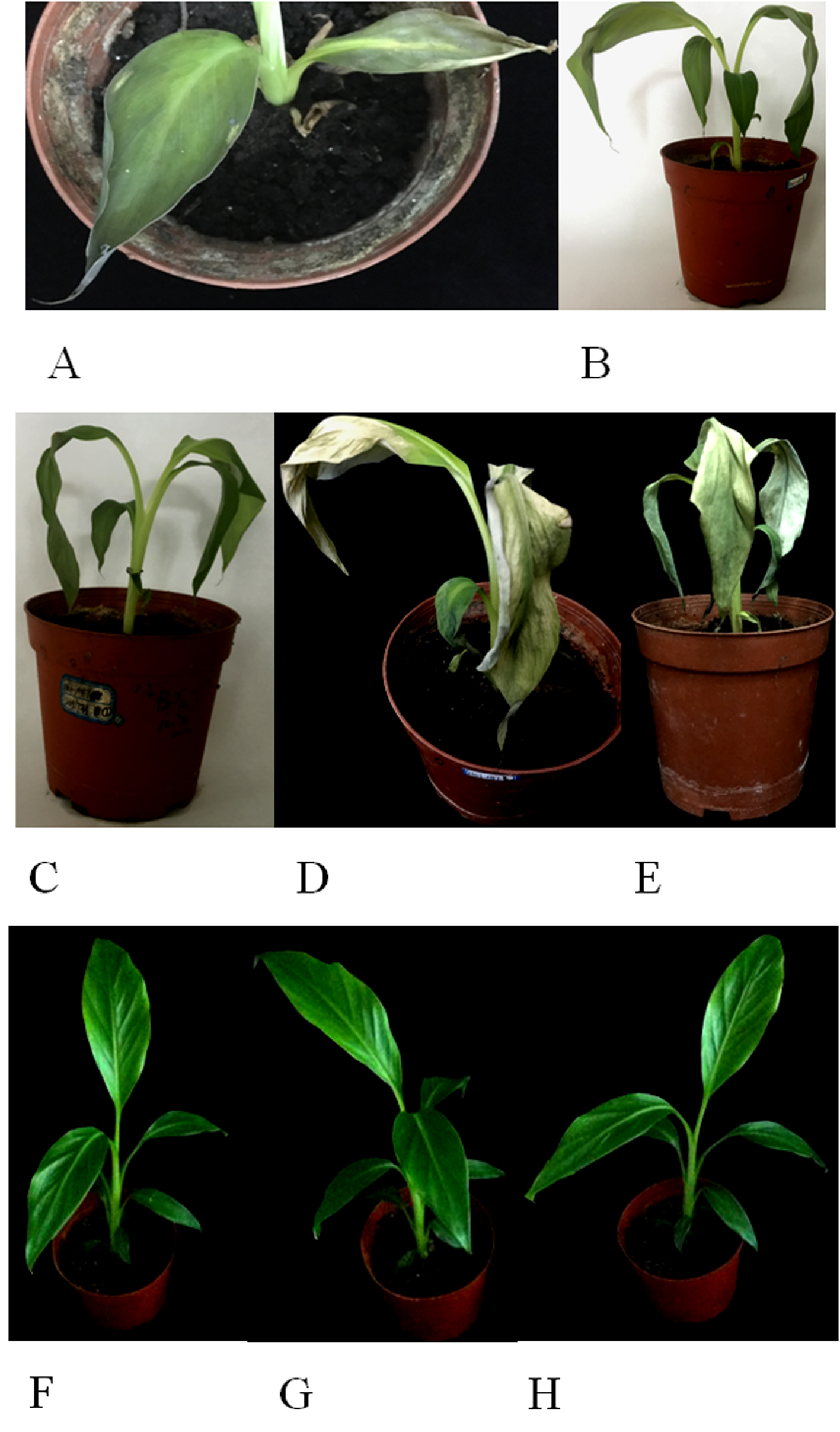

Supplement: S2 Fig — A, ‘Tianbaojiao’ at 4°C, 3 h; B, ‘Tianbaojiao’ at 4°C, 5 h; C, ‘Tianbaojiao’ at 4°C, 7 h; D-E, 4°C stressed ‘Tianbaojiao’ at 28°C to recover; F, the wild banana at 4°C, 3 h; G, the wild banana at 4°C, 5 h; H, the wild banana at 4°C, 7 h. (TIF) [file pone.0200149.s002.tif]

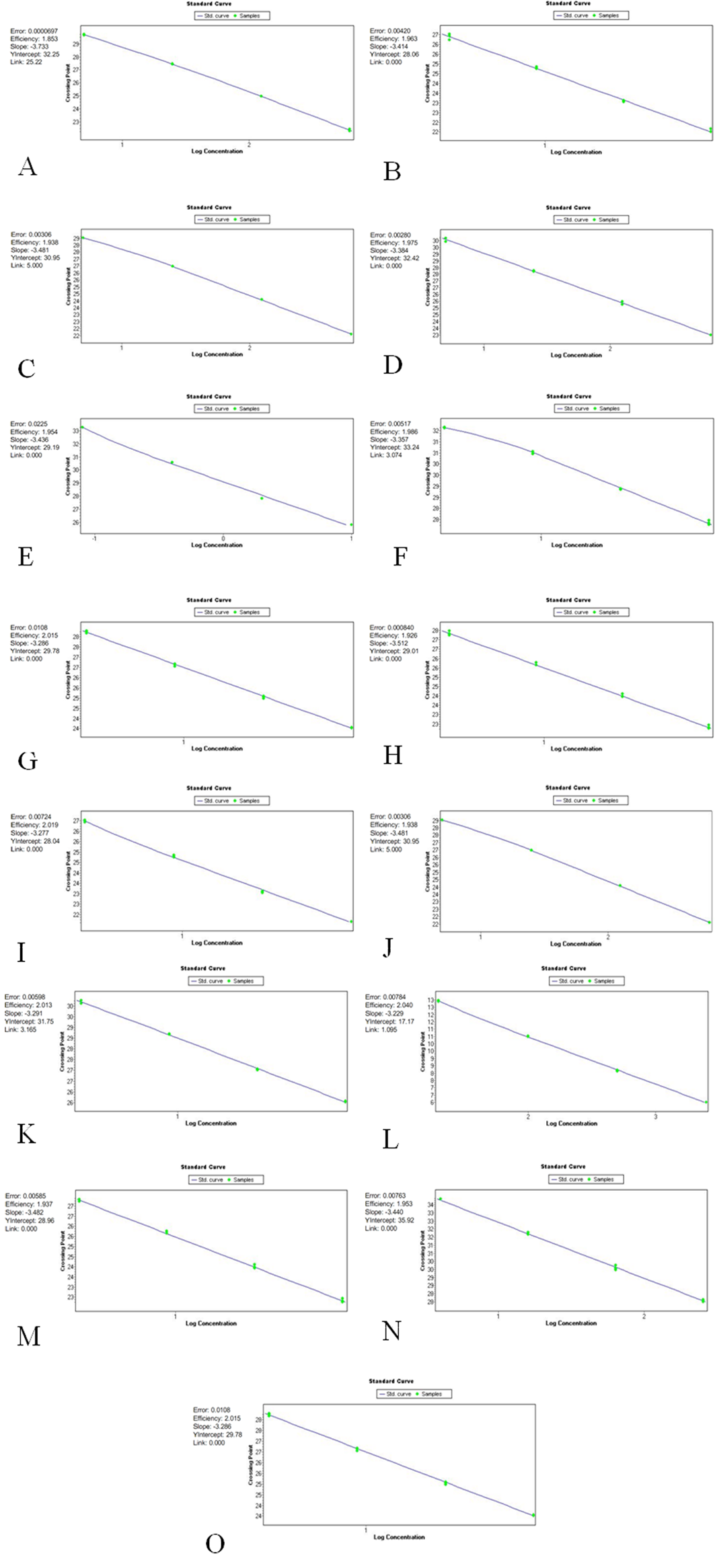

Supplement: S3 Fig — The amplification efficiency for each primer pairs of the CKII family genes and CAC was determined in a qPCR assay using a five-fold dilution series from a pooled cDNA template. A, CKIIα-1; B, CKIIβ-4-1; C, CKIIβ-like-1; D, CKIIβ-4-2; E, CKIIβ-4-3; F, CKIIβ-3-like; G, CKIIβ-4-4; H, CKIIα-2; I, CKIIα-3; J, CKIIα-4; K, CKIIβ-like-2a; L, CKIIβ-like-2b; M, CKIIα-5; N, CKIIβ-like-3; O, CAC. (TIF) [file pone.0200149.s003.tif]

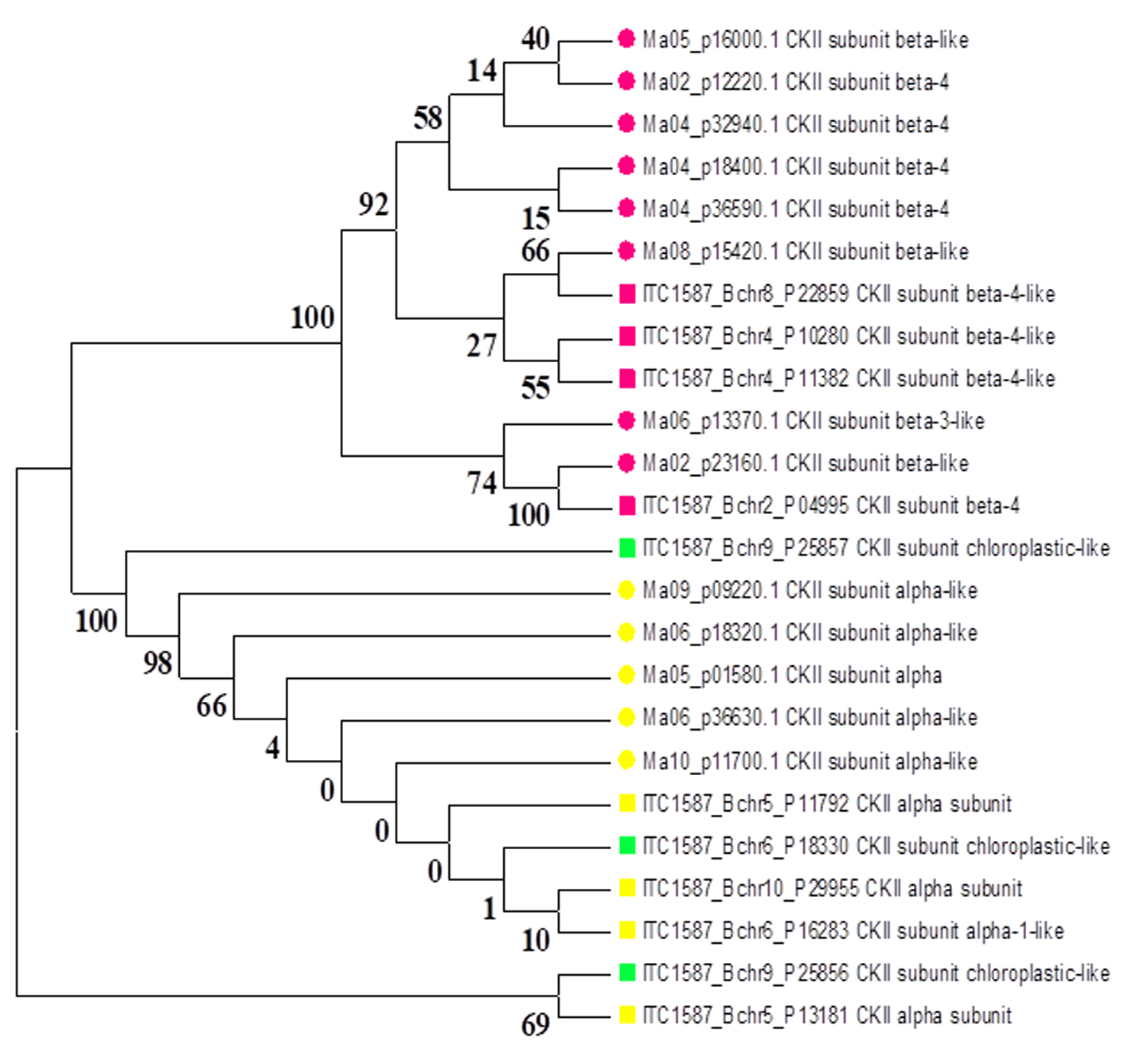

Supplement: S4 Fig — Phylogenetic analysis of the CKII family members in banana genome A and B. The phylogenetic tree was constructed using MEGA5 by the neighbor-joining (NJ) method and 1000 bootstrap replicates. The tree was divided into two phylogenetic subgroups. The CKII β members of the two Musa plants were assigned to one branch, and the CKII α members combined with unclassified subunit CKII members were assigned to another branch. (TIF) [file pone.0200149.s004.tif]

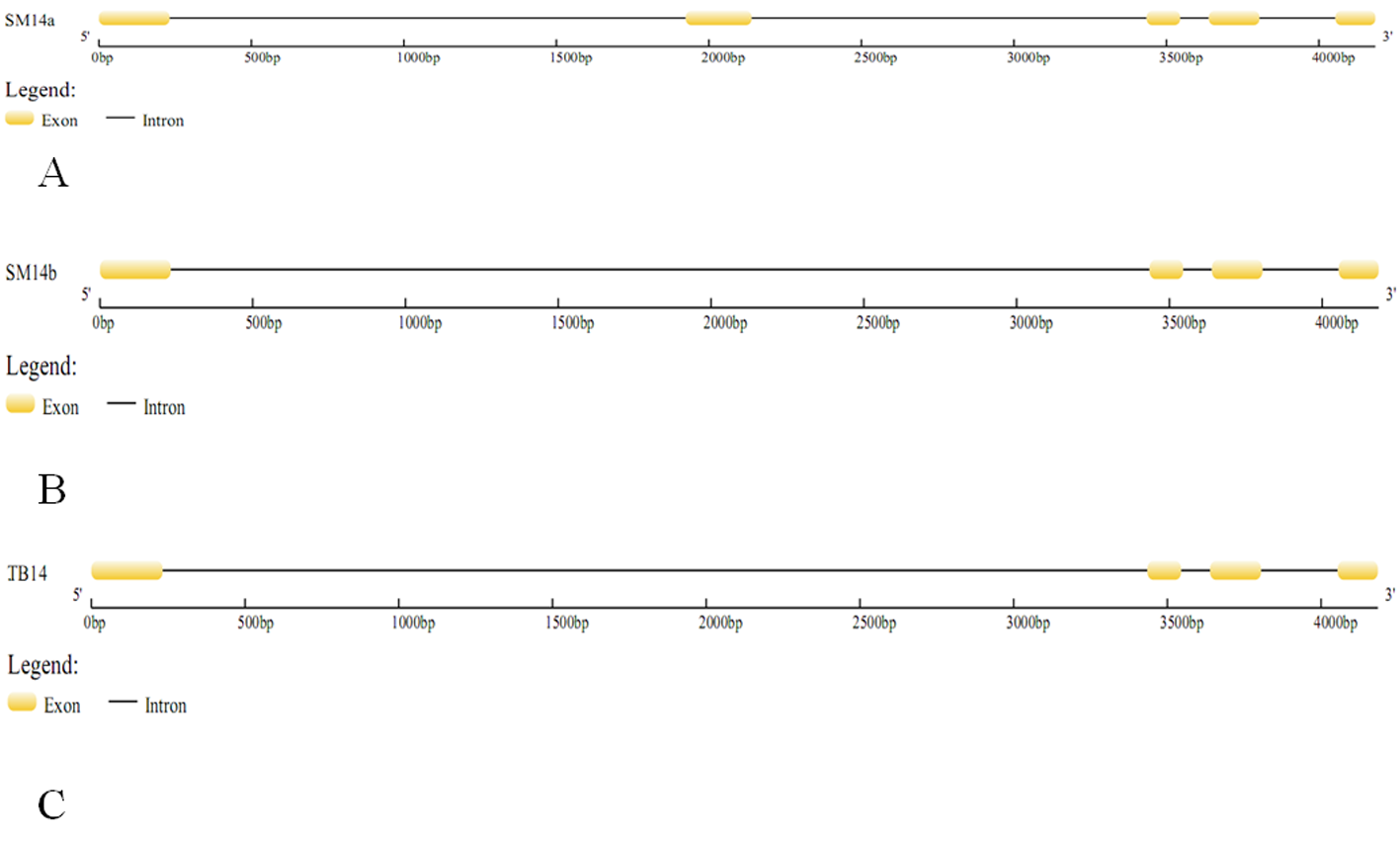

Supplement: S5 Fig — Structural analyses of CKIIβ-like-2a, CKIIβ-like-2b in wild banana and CKIIβ-like-2 in ‘Tianbaojiao’ were performed using GSDS showing the CKIIβ-like-2b in wild banana and CKIIβ-like-2 in ‘Tianbaojiao’ might be the exon deletion alternative splicing transcript. The exons and introns are represented by colored boxes and black lines, respectively. A, the gene structure of MiCKIIβ-like-2a; B, the gene structure of MiCKIIβ-like-2b; C, the gene structure of MaCKIIβ-like-2. The wild banana and ‘Tianbaojiao’ were abbreviated as ‘SM’ and ‘TB’. CKIIβ-like-2 was abbreviated as ‘14’. (TIF) [file pone.0200149.s005.tif]

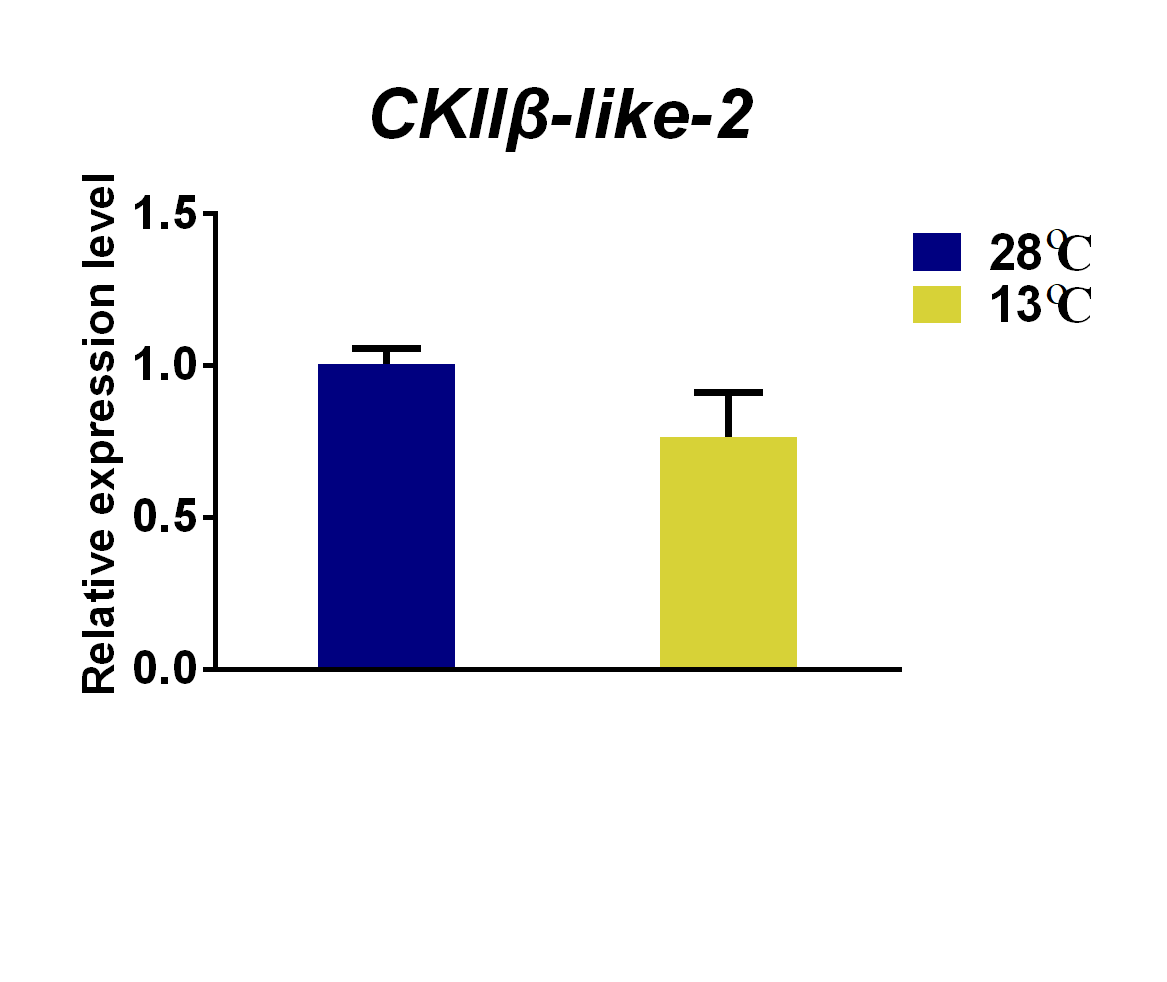

Supplement: S6 Fig — Transcripts abundance was quantified using qRT-PCR. The expression levels from three independent biological replicates were analyzed. (TIF) [file pone.0200149.s006.tif]
