# Supplementary material for: Genome-wide identification and characterization of the CKII gene family in the cultivated banana cultivar (Musa spp. cv Tianbaojiao) and the wild banana (Musa itinerans)
Source: PLoS One. 2018 Jul 11;13(7):e0200149. doi: 10.1371/journal.pone.0200149 (PMC6040749; doi:10.1371/journal.pone.0200149)
Supplement: S1 Table — ‘Tianbaojiao’ and the wild banana were abbreviated as ‘TB’ and ‘SM’. The CKII gene family members of CKIIα-1, CKIIβ-4-1, CKIIβ-like-1, CKIIβ-4-2, CKIIβ-4-3, CKIIβ-3-like, CKIIβ-4-4, CKIIα-2, CKIIα-3, CKIIα-4, CKIIβ-like-2a, CKIIβ-like-2b, CKIIα-5, CKIIβ-like-3, were abbreviated CKII-1, CKII-2, CKII-3, CKII-4, CKII-5, CKII-6, CKII-7, CKII-9, CKII-10, CKII-13, CKII-14a, CKII-14b, CKII-15 and CKII-16. (DOC) [file pone.0200149.s007.doc]

**S1 Table The primers used for gene cloning in this study**

| **Primer pairs**  **name** | **Primer sequences ( 5′** - **3′ )** | **Target** [**fragment**](javascript:void(0);) | **Annealing**  **temperature (℃)** | **Annealing**  **time** |
| --- | --- | --- | --- | --- |
| TB-CKII-1-F  TB-CKII-1-R | ATGTCGAAAGCCCGGGTATAT  TTATTGGGTCCTCATCCTGCTAT | TB-CKII-1 | 51 | 1 min |
| TB-CKII-2-F  TB-CKII-2-R | CGAGTGAATTGGGGAGGAGAG  CTATTCATCTGGCGGCTCAAC | TB-CKII-2 | 54 | 1 min |
| TB-CKII-3-F  TB-CKII-3-R | CCCTAAGACTTCTTTAATGGTTGC  CTCCGAGCAATTATTTAGAGCCCT | TB-CKII-3 | 53 | 1 min |
| TB-CKII-4-F  TB-CKII-4-R | ATGCATAGGGATCGGGGAGG  TGACCCTTCCATGTGACAGAT | TB-CKII-4 | 52 | 1 min |
| TB-CKII-5-F  TB-CKII-5-R | GAGGGAGATATGTATAGAGAGAG  TGGCTCAACCTCCATTGTCAT | TB-CKII-5 | 51 | 1 min |
| TB-CKII-6-F  TB-CKII-6-R | ATGTTGAAGACCGGATCTAC  CTACAAGGTATTAGTCGATGTGT | TB-CKII-6 | 50 | 1 min |
| TB-CKII-7-F  TB-CKII-7-R | CGAAGAGGAGGGAGATATGTAT  CCGCTCAACTCCACTGTCAT | TB-CKII-7 | 52 | 1 min |
| TB-CKII-9-F  TB-CKII-9-R | CTTCCTATTTCTATGGCCTTTGG  GAACTTCAAGTACGAGCCCTG | TB-CKII-9 | 52 | 1 min  30 s |
| TB-CKII-10-F  TB-CKII-10-R | ATGTCGAAAGCCCGGGTATAC  ACTTTTACTGGGTCCGCATTCT | TB-CKII-10 | 52 | 1 min |
| TB-CKII-13-F  TB-CKII-13-R | CTTCCTATTTCTATGGCCTTTGG  CTAAGCATGAGTTCTGCTGCTT | TB-CKII-13 | 52 | 1min 30 s |
| TB-CKII-14-F  TB-CKII-14-R | CTCATTCGATTTGTTTAGCTGCAT  AGTTCCTCGATACACCCCTTTC | TB-CKII-14 | 51 | 1 min |
| TB-CKII-15-F  TB-CKII-15-R | GAATGAGAGATGCCCCCGAT  AATATTACTGGGTGCGAGTCCT | TB-CKII-15 | 52 | 1 min |
| TB-CKII-16-F  TB-CKII-16-R | ATGTATAGAGAGAGGGGAGGT  TCTCTTTGTTTGGCAGCTCAG | TB-CKII-16 | 51 | 1 min |
| TB-CKII-14-F  TB-CKII-14-R | CTCATTCGATTTGTTTAGCTGCAT  AGTTCCTCGATACACCCCTTTC | TB-CKII-14-gDNA | 51 | 3 min |
| SM-CKII-1-F  SM-CKII-1-R | ATGTCGAAAGCCCGGGTATAT  TTATTGGGTCCTCATCCTGCTAT | SM-CKII-1 | 51 | 1 min |
| SM-CKII-2-F  SM-CKII-2-R | CGAGTGAATTGGGGAGGAGAG  CTATTCATCTGGCGGCTCAAC | SM-CKII-2 | 54 | 1 min |
| SM-CKII-3-F  SM-CKII-3-R | CCCTAAGACTTCTTTAATGGTTGC  CTCCGAGCAATTATTTAGAGCCCT | SM-CKII-3 | 53 | 1 min |
| SM-CKII-4-F  SM-CKII-4-R | ATGCATAGGGATCGGGGAGG  TGACCCTTCCATGTGACAGAT | SM-CKII-4 | 52 | 1 min |
| SM-CKII-5-F  SM-CKII-5-R | GAGGGAGATATGTATAGAGAGAG  TGGCTCAACCTCCATTGTCAT | SM-CKII-5 | 51 | 1 min |
| SM-CKII-6-F  SM-CKII-6-R | ATGTTGAAGACCGGATCTAC  CTACAAGGTATTAGTCGATGTGT | SM-CKII-6 | 50 | 1 min |
| SM-CKII-7-F  SM-CKII-7-R | CGAAGAGGAGGGAGATATGTAT  CCGCTCAACTCCACTGTCAT | SM-CKII-7 | 52 | 1 min |
| SM-CKII-9-F  SM-CKII-9-R | TTCCCTTCTCGGTTCCTATTTCTATG  TTGGCCTGTCCTGGTGATCGTACT | SM-CKII-9 | 56 | 1 min |
| SM-CKII-10-F  SM-CKII-10-R | ATGTCGAAAGCCCGGGTATAC  ACTTTTACTGGGTCCGCATTCT | SM-CKII-10 | 52 | 1 min |
| SM-CKII-13-F  SM-CKII-13-R | CTTCCTATTTCTATGGCCTTTGG  CGGGATTCATCACTCGGATTTC | SM-CKII-13 | 51 | 1 min  18 s |
| SM-CKII-14-F  SM-CKII-14-R | CTCATTCGATTTGTTTAGCTGCAT  AGTTCCTCGATACACCCCTTTC | SM-CKII-14 | 51 | 1 min |
| SM-CKII-15-F  SM-CKII-15-R | GAATGAGAGATGCCCCCGAT  AATATTACTGGGTGCGAGTCCT | SM-CKII-15 | 52 | 1 min |
| SM-CKII-16-F  SM-CKII-16-R | ATGTATAGAGAGAGGGGAGGT  TCTCTTTGTTTGGCAGCTCAG | SM-CKII-16 | 51 | 1 min |
| SM-CKII-14-F  SM-CKII-14-R | CTCATTCGATTTGTTTAGCTGCAT  AGTTCCTCGATACACCCCTTTC | SM-CKII-14-gDNA | 51 | 3 min |

‘Tianbaojiao’ and the wild banana were abbreviated as ‘TB’ and ‘SM’. The *CKII* gene family members of *CKIIα-1*, *CKIIβ-4-1*, *CKIIβ-like-1*, *CKIIβ-4-2*, *CKIIβ-4-3*, *CKIIβ-3-like*, *CKIIβ-4-4*, *CKIIα-2*, *CKIIα-3*, *CKIIα-4*, *CKIIβ-like-2a*, *CKIIβ-like-2b*, *CKIIα-5*, *CKIIβ-like-3*, were abbreviated *CKII-1*, *CKII-2*, *CKII-3*, *CKII-4*, *CKII-5*, *CKII-6*, *CKII-7*, *CKII-9*, *CKII-10*, *CKII-13*, *CKII-14a*, *CKII-14b*, *CKII-15* and *CKII-16*.
