# Supplementary material for: Genome-wide identification and characterization of the CKII gene family in the cultivated banana cultivar (Musa spp. cv Tianbaojiao) and the wild banana (Musa itinerans)
Source: PLoS One. 2018 Jul 11;13(7):e0200149. doi: 10.1371/journal.pone.0200149 (PMC6040749; doi:10.1371/journal.pone.0200149)
Supplement: S2 Table — The CKII gene family members of CKIIα-1, CKIIβ-4-1, CKIIβ-like-1, CKIIβ-4-2, CKIIβ-4-3, CKIIβ-3-like, CKIIβ-4-4, CKIIα-2, CKIIα-3, CKIIα-4, CKIIβ-like-2a, CKIIβ-like-2b, CKIIα-5, CKIIβ-like-3, were abbreviated CKII-1, CKII-2, CKII-3, CKII-4, CKII-5, CKII-6, CKII-7, CKII-9, CKII-10, CKII-13, CKII-14a, CKII-14b, CKII-15 and CKII-16. (DOC) [file pone.0200149.s008.doc]

**S2 Table** The primers used for qPCR assay in this study

| **Primer pairs name** | **Primer sequences ( 5′-3′ )** | **Ampliﬁcation efﬁciency** |
| --- | --- | --- |
| qPCR-CKII-1-F  qPCR-CKII-1-R | ATGTAACCAATAATGAGCGATGC  GTCATAATCCGTCAATGTAGGGT | 1.853 |
| qPCR-CKII-2-F  qPCR-CKII-2-R | GAACAGCAAGGAGAAGGATAGG  TGTACTCATCATCAACTTCGCAG | 1.963 |
| qPCR-CKII-3-F  qPCR-CKII-3-R | GCAAGCCAACTCCAGAACTATC  GCCACTAAACGAGTCATTGTCC | 1.938 |
| qPCR-CKII-4-F  qPCR-CKII-4-R | GCGTTCTGCTTCTCTTTCCA  TCAGTAAACAAATCACCATGGG | 1.975 |
| qPCR-CKII-5-F  qPCR-CKII-5-R | TCCAAGAACAAATGCTCAGATG  ACTCAACATCCAGAATCAGATCG | 1.954 |
| qPCR-CKII-6-F  qPCR-CKII-6-R | GCTTGCTGAAATTGGGAGTG  CCTGATTATGTTCTTCGCCATC | 1.986 |
| qPCR-CKII-7-F  qPCR-CKII-7-R | AGCAGCAAGGAGAAGGATAGG  TGTATTCATCATCAACTTCGCAG | 2.015 |
| qPCR-CKII-9-F  qPCR-CKII-9-R | CACCTCGCCACCTACTGC  TCGTAATCCCAGTAATCTTTGG | 1.926 |
| qPCR-CKII-10-F  qPCR-CKII-10-R | TGAACGGTGCATTATCAAGATC  TCATAATCCGACAGTGTAGGGTAC | 2.019 |
| qPCR-CKII-13-F  qPCR-CKII-13-R | CCACTTCGCCTCTTCCAC  TCGTAGTCCCAGTAATCCTTGG | 1.938 |
| qPCR-CKII-14a-F  qPCR-CKII-14a-R | CAAGAACAAGTGCTCCGATG  CAAGAATTAGATCGAGAGCACAG | 2.013 |
| qPCR-CKII-14b-F  qPCR-CKII-14b-R | GGATTCATCGTGCCAAAAG  TCAGTAAACATATCGCCATCG | 2.040 |
| qPCR-CKII-15-F  qPCR-CKII-15-R | GTTGTTCGAAAGGTTGGCAG  GGAGTTTTTGAATGCTGGTCC | 1.937 |
| qPCR-CKII-16-F  qPCR-CKII-16-R | GTATAGAGAGAGGGGAGGTTGC  TGCTCTTGGACAAGGTTCG | 1.953 |
| CAC-F  CAC-R | CTCCTATGTTGCTCGCTTATG  GGCTACTACTTCGGTTCTTTC | 2.015 |

The *CKII* gene family members of *CKIIα-1*, *CKIIβ-4-1*, *CKIIβ-like-1*, *CKIIβ-4-2*, *CKIIβ-4-3*, *CKIIβ-3-like*, *CKIIβ-4-4*, *CKIIα-2*, *CKIIα-3*, *CKIIα-4*, *CKIIβ-like-2a*, *CKIIβ-like-2b*, *CKIIα-5*, *CKIIβ-like-3*, were abbreviated *CKII-1*, *CKII-2*, *CKII-3*, *CKII-4*, *CKII-5*, *CKII-6*, *CKII-7*, *CKII-9*, *CKII-10*, *CKII-13*, *CKII-14a*, *CKII-14b*, *CKII-15* and *CKII-16*.
