# Supplementary material for: Genome-wide identification and characterization of the CKII gene family in the cultivated banana cultivar (Musa spp. cv Tianbaojiao) and the wild banana (Musa itinerans)
Source: PLoS One. 2018 Jul 11;13(7):e0200149. doi: 10.1371/journal.pone.0200149 (PMC6040749; doi:10.1371/journal.pone.0200149)
Supplement: S3 Table — ‘Ser’ and ‘Thr’ were abbreviated as ‘S’ and ‘T’. (DOC) [file pone.0200149.s009.doc]

**S3 Table Predict of the CKII phosphorylation sites among the wild banana, ‘Tianbaojiao’ and genome A**

|  | **Protein name** | **Length** | **NO.** | **phosphorylation sites/amino acid residue of phosphorylation** |
| --- | --- | --- | --- | --- |
| **Banana**  **genome**  **A** | Ma06_p18320.1 | 333 | 2 | 46/S; 55/T |
|  | Ma04_p36590.1 | 280 | 12 | 10/S; 71/S; 75/S; 77/T; 79/S; 82/S; 85/S; 87/S; 143/S; 148/T; 150/T; 159/S |
|  | Ma02_p23160.1 | 226 | 8 | 20/S; 27/T; 30/S; 31/T; 32/S; 90/S; 99/S; 181/S |
|  | Ma04_p18400.1 | 286 | 9 | 10/S; 78/S; 81/S; 83/T; 85/S; 88/S; 91/S; 93/S; 156/T |
|  | Ma02_p12220.1 | 279 | 10 | 71/S; 74/S; 76/T; 78/S; 81/S; 84/S; 86/S; 142/S; 149/T; 158/S |
|  | Ma06_p13370.1 | 290 | 7 | 78/S; 81/T; 84/S; 86/S; 144/S; 153/S; 157/S |
|  | Ma04_p32940.1 | 280 | 11 | 10/S; 71/S; 75/S; 77/T; 79/S; 82/S; 85/S; 87/S; 143/S; 150/T; 159/S |
|  | Ma09_p09220.1 | 416 | 2 | 129/S; 138/T |
|  | Ma10_p11700.1 | 333 | 2 | 46/S; 55/T |
|  | Ma06_p36630.1 | 404 | 2 | 118/S; 127/T |
|  | Ma08_p15420.1 | 284 | 9 | 76/S; 82/T; 84/S; 87/S; 90/S; 92/S; 96/T; 154/T; 163/S |
|  | Ma05_p01580.1 | 391 | 2 | 104/S; 113/S |
|  | Ma05_p16000.1 | 280 | 10 | 10/S; 71/S; 77/T; 79/S; 82/S; 85/S; 87/S; 143/S; 150/T; 159/S |
| **‘Tianbao**  **jiao’** | CKII α-1 | 333 | 2 | 46/S; 55/T |
|  | CKII β-4-1 | 280 | 11 | 10/S; 71/S; 75/S; 77/T; 79/S; 82/S; 85/S; 87/S; 143/S; 150/T; 159/S |
|  | CKII β-like-1 | 226 | 8 | 22/T; 27/T; 30/S; 31/T; 32/S; 90/S; 99/S; 181/S |
|  | CKII β-4-2 | 285 | 11 | 10/S; 76/S; 79/S; 81/T; 83/S; 86/S; 89/S; 91/S; 147/S; 154/T; 163/S |
|  | CKII β-4-3 | 280 | 10 | 71/S; 75/S; 77/T; 79/S; 82/S; 85/S; 87/S; 143/S; 150/T; 159/S |
|  | CKII β-3-like | 290 | 7 | 78/S; 81/T; 84/S; 86/S; 144/S; 153/S; 157/S |
|  | CKII β-4-4 | 280 | 11 | 10/S; 71/S; 75/S; 77/T; 79/S; 82/S; 85/S; 87/S; 143/S; 150/T; 159/S |
|  | CKII α-2 | 416 | 2 | 129/S; 138/T |
|  | CKII α-3 | 333 | 2 | 46/S; 55/T |
|  | CKII α-4 | 404 | 2 | 118/S; 127/T |
|  | CKII β-like-2 | 212 | 2 | 82/T; 91/S |
|  | CKII α-5 | 391 | 1 | 113/S |
|  | CKII β-like-3 | 280 | 10 | 71/S; 75/S; 77/T; 79/S; 82/S; 85/S; 87/S; 143/S; 150/T; 159/S |
| **The**  **wild**  **banana** | CKII α-1 | 333 | 2 | 46/S; 55/T |
|  | CKII β-4-1 | 280 | 11 | 10/S; 71/S; 75/S; 77/T; 79/S; 82/S; 85/S; 87/S; 143/S; 150/T; 159/S |
|  | CKII β-like-1 | 226 | 9 | 20/S; 22/T; 27/T; 30/S; 31/T; 32/S; 90/S; 99/S; 181/S |
|  | CKII β-4-2 | 286 | 10 | 10/S; 78/S; 83/T; 85/S; 88/S; 91/S; 93/S; 149/S; 156/T; 165/S |
|  | CKII β-4-3 | 279 | 11 | 10/S; 71/S; 74/S; 76/T; 78/S; 81/S; 84/S; 86/S; 142/S; 149/T; 158/S |
|  | CKII β-3-like | 280 | 5 | 78/S; 81/T; 84/S; 86/S; 157/S |
|  | CKII β-4-4 | 280 | 11 | 10/S; 71/S; 75/S; 77/T; 79/S; 82/S; 85/S; 87/S; 143/S; 150/T; 159/S |
|  | CKII α-2 | 416 | 2 | 129/S; 138/T |
|  | CKII α-3 | 333 | 2 | 46/S; 55/T |
|  | CKII α-4 | 408 | 2 | 118/S; 127/T |
|  | CKII β-like-2a | 284 | 8 | 76/S; 82/T; 84/S; 87/S; 90/S; 92/S; 154/S; 163/S |
|  | CKII β-like-2b | 212 | 2 | 82/T; 91/S |
|  | CKII α-5 | 391 | 1 | 113/S |
|  | CKII β-like-3 | 280 | 11 | 10/S; 71/S; 75/S; 77/T; 79/S; 82/S; 85/S; 87/S; 143/S; 150/T; 159/S |

‘Ser’ and ‘Thr’ were abbreviated as ‘S’ and ‘T’.
